# Bioengineered 3D models of human pancreatic cancer recapitulate in vivo tumour biology

**DOI:** 10.1038/s41467-021-25921-9

**Published:** 2021-09-24

**Authors:** David Osuna de la Peña, Sara Maria David Trabulo, Estelle Collin, Ying Liu, Shreya Sharma, Marianthi Tatari, Diana Behrens, Mert Erkan, Rita T. Lawlor, Aldo Scarpa, Christopher Heeschen, Alvaro Mata, Daniela Loessner

**Affiliations:** 1 Barts Cancer Institute, Queen Mary University of London, London, UK; 2 Institute of Bioengineering, Queen Mary University of London, London, UK; 3 EPO – Experimental Pharmacology and Oncology GmbH, Berlin, Germany; 4 Department of Surgery, Koç University School of Medicine, Istanbul, Turkey; 5 Koç University Translational Research Center – KUTTAM, Istanbul, Turkey; 6 Department of Diagnostics and Public Health, Section of Pathology, University of Verona, Verona, Italy; 7 ARC-Net, Applied Research on Cancer Centre, University of Verona, Verona, Italy; 8 Center for Single-Cell Omics, Shanghai Jiao Tong University School of Medicine, Shanghai, China; 9 Laboratory of Pancreatic Cancer Heterogeneity, Candiolo Cancer Institute, FPO-IRCCS, Candiolo, Turin, Italy; 10 School of Pharmacy, University of Nottingham, Nottingham, UK; 11 Department of Chemical and Environmental Engineering, University of Nottingham, Nottingham, UK; 12 Biodiscovery Institute, University of Nottingham, Nottingham, UK; 13 Department of Chemical Engineering, Faculty of Engineering, Monash University, Melbourne, VIC Australia; 14 Department of Materials Science and Engineering, Faculty of Engineering, Monash University, Melbourne, VIC Australia; 15 Department of Anatomy and Developmental Biology, Faculty of Medicine, Nursing and Health Sciences, Monash University, Melbourne, VIC Australia; 16 Present Address: Lungs for Living Research Centre, UCL Respiratory, University College London, London, UK; 17 Present Address: Faculty of Infectious and Tropical Diseases, London School of Hygiene and Tropical Medicine, University of London, London, UK

**Keywords:** Cancer microenvironment, Cancer models, Cancer stem cells

## Abstract

Patient-derived in vivo models of human cancer have become a reality, yet their turnaround time is inadequate for clinical applications. Therefore, tailored ex vivo models that faithfully recapitulate in vivo tumour biology are urgently needed. These may especially benefit the management of pancreatic ductal adenocarcinoma (PDAC), where therapy failure has been ascribed to its high cancer stem cell (CSC) content and high density of stromal cells and extracellular matrix (ECM). To date, these features are only partially reproduced ex vivo using organoid and sphere cultures. We have now developed a more comprehensive and highly tuneable ex vivo model of PDAC based on the 3D co-assembly of peptide amphiphiles (PAs) with custom ECM components (PA-ECM). These cultures maintain patient-specific transcriptional profiles and exhibit CSC functionality, including strong in vivo tumourigenicity. User-defined modification of the system enables control over niche-dependent phenotypes such as epithelial-to-mesenchymal transition and matrix deposition. Indeed, proteomic analysis of these cultures reveals improved matrisome recapitulation compared to organoids. Most importantly, patient-specific in vivo drug responses are better reproduced in self-assembled cultures than in other models. These findings support the use of tuneable self-assembling platforms in cancer research and pave the way for future precision medicine approaches.

## Abstract (web summary)

Personalized cancer medicine currently lacks custom platforms that mimic the microenvironment of human tissues. Here, the authors show how self-assembled patient-derived models of pancreatic cancer recapitulate key biological features of the original tumours such as matrix composition and stemness.

## Introduction

With a 5-year survival of 10%, pancreatic ductal adenocarcinoma (PDAC) remains a leading cause of cancer-related death worldwide^1^. The intrinsic resistance of PDAC tumours is compounded by intratumour and interpatient heterogeneity, as well as extrinsic factors, such as pronounced desmoplasia and hypovascularization, which limit the efficacy of existing treatments, such as gemcitabine, nab-paclitaxel and FOLFIRINOX^2^. The development of more effective therapies, tailored to individual patients, may benefit from the use of platforms that incorporate patient-derived cells for predictive drug testing. Patient-derived xenografts (PDX) in mice have shown great promise in their ability to reproduce patient response^3^, but their establishment takes months, which hampers their use in the context of PDAC, where median overall survival is only 6 months. Thus, faster and more scalable ex vivo platforms are required for the establishment of clinically useful patient-specific models of pancreatic cancer.

Current ex vivo platforms allow cell cultures in 3D, either floating or embedded in biomimetic matrices. Floating spheroid cultures are a simple and popular 3D approach^4^. However, the lack of matrix attachment, artificially high nutrient gradients, variable growth patterns and limited control over cell distribution make nonadherent spheroid cultures less desirable than matrix-based approaches like Matrigel, decellularized tissues or custom hydrogels^5^. Matrigel, although routinely used for organoid and organotypic cultures^6^, is limited by its murine origin, undefined composition and batch-to-batch variability^7^. Similarly, decellularized tissues suffer from high complexity and poor tractability^8^. In contrast, hydrogels based on synthetic polymers, such as polyethylene glycol, offer great flexibility in the control of their physicochemical properties^9^, while being less biocompatible than hydrogels based on natural materials. Ex vivo platforms based on hybrid biomaterials, such as methacrylated hyaluronan^10^ and gelatin methacryloyl^11^, have shown excellent physical tuneability, but have limitations in mimicking biological signals other than hyaluronan and gelatin (denatured collagen). Advances in biofabrication, such as 3D printing^12^ and microfluidic systems^13^ have enabled control over PDAC culture microarchitecture and fluid flow, respectively. Nevertheless, these models still lack the capacity to recreate the diverse and dense PDAC stroma at the nanoscale with custom physical properties and composition.

More recently, various types of self-assembling peptides have been developed for the ex vivo modelling of tissues with enhanced versatility^14–16^. These peptides offer the possibility to bioengineer complex microenvironments through a reductionist approach, controlling nanoscale geometries and the presentation of epitopes to selectively signal cells^17^. Peptide amphiphiles (PAs) are a class of self-assembling peptides capable of generating nanofibrous hydrogels that can recreate the architecture of the natural extracellular matrix (ECM)^18^. PAs can be designed to bind selectively and nonselectively to molecules while assembling into high-aspect-ratio cylindrical micelles (nanofibres) in polar solutions. To enhance the molecular complexity and biological relevance of these matrices, we have established methodologies to use PAs to co-assemble with and organise ECM macromolecules and other proteins, such as keratins^19^ into hydrogel matrices with tuneable signalling capabilities^20^, structures^21,22^ and physical properties^23^. Notably, recreation of functional tumour niches requires a more complex, diverse and dynamic organisation of ECM components, which is not currently attainable with most ex vivo tumour models (Supplementary Table 1).

In this work, we use a multicomponent self-assembling approach to establish an instructive matrix (PA-ECM) composed of PA molecules and multiple ECM components of PDAC, including collagen type I, fibronectin, laminin and hyaluronan. Through the immediate presentation of these macromolecules as both the structural and signalling components of the hydrogel, we expect to promote niche-dependent phenotypes associated with poor prognosis, including cancer stem cell (CSC) propagation, epithelial-to-mesenchymal transition (EMT) and ECM deposition. We hypothesise that a CSC-supportive environment will enable a more faithful approximation of the patients’ PDAC characteristics than directed differentiation via growth factors (as in organoids). To recapitulate the tumour microenvironment, we include patient-derived pancreatic stellate cells (PSCs) and primary macrophages, which are the key cellular components of the PDAC stroma^24^. As biological references for the validation of our self-assembling platform, we use patient-matched tumour tissue and PDXs, and we also benchmark our findings against patient-matched 2D monolayers, Matrigel-embedded organoids and sphere cultures.

## Results

### Construction of co-assembled PA-ECM matrices for PDAC cell culture

**Fig. 1:**
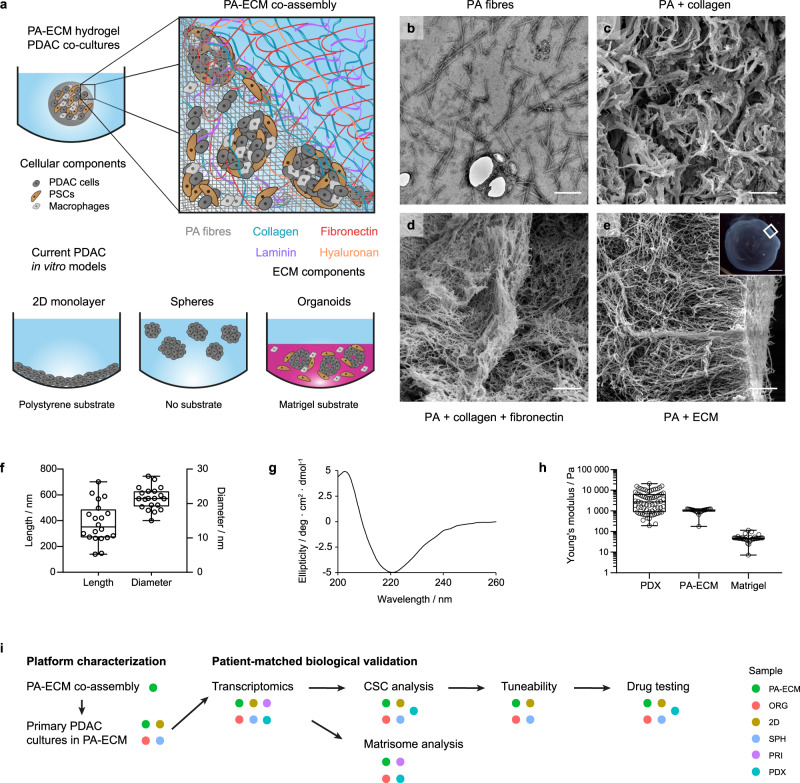
Design and characterisation of PA-ECM co-assembling matrices. **a** Schematic illustration of PA hydrogel co-assembly with ECM macromolecules compared to currently used substrates for the in vitro modelling of PDAC. **b** Transmission electron micrograph of PA fibres. Scale bar: 100 µm. **c**–**e** Scanning electron micrographs of PA hydrogels co-assembled with collagen (c), collagen and fibronectin (d) and all ECM components (e); insert indicates imaged area within a 2 µL hydrogel. Scale bar: 2 µm. Insert scale bar: 500 µm. **f** PA fibre size measured from TEM micrographs. Box plots indicate range, interquartile range and median. *n* = 20 individual fibres. **g** PA fibre circular dichroism spectrum in HEPES buffer. **h** Stiffness (Young’s modulus) of PDX tissue, as measured by atomic force microscopy (*n* = 71 measurements across a tissue section), compared to PA-ECM (*n* = 24 measurements of a 10 mg/mL gel) and Matrigel (*n* = 38 measurements of a 10 mg/mL gel), as measured by rheometry. Box plots indicate range, interquartile range and median. **i** Experimental outline for the validation of the platform as a model for pancreatic cancer.

While current in vitro models of PDAC are characterised by a relatively simple design that restricts control over the physical properties and composition of the microenvironment, PA molecules can be co-assembled with ECM macromolecules to form tuneable matrices for cell culture (Fig. 1a). The fibrillar nature of this model was verified by electron microscopy (Fig. 1b–e). Upon co-assembly, PA fibres form highly aligned bundles whose arrangement varies according to the composition of the ECM: PA-collagen hydrogels exhibit thicker fibrils (Fig. 1c), while the addition of fibronectin results in thinner, more aligned mesh-like structures (Fig. 1d). Similar structures were observed when PAs are co-assembled with collagen, fibronectin, laminin and hyaluronan (Fig. 1e), which we subsequently used as our standard PA-ECM hydrogels. On average, PA fibres were approximately 20 × 300 nm in size (Fig. 1f), similar to most ECM proteins like collagen^25^. The β-sheet conformation of the assembled fibres was confirmed by circular dichroism spectroscopy (Fig. 1g). In terms of stiffness, the Young’s modulus of PA-ECM co-assembled hydrogels was around 1 kPa, which is within the range of pancreatic PDX tissues (Fig. 1h), as well as published data for primary PDAC^26^, while Matrigel, used for the generation of organoids, was much softer than PDAC tissue at around 90 Pa. Due to the heterogeneous nature of the PDAC stroma, some tumour areas exhibit a higher stiffness of up to 20 kPa. This can be recreated in the PA-ECM system by modifying peptide or gelling agent (e.g., collagen or CaCl_2_) concentration, PA-macromolecule affinity^19^ or the peptide sequence^27^. Furthermore, given the inherent anisotropy of PDAC stroma, mechanical properties will depend on the type of measurement performed (e.g., tension, compression). In this study, we have conducted indentation measurements via an atomic force microscope as it has been used extensively to describe mechanical properties of tumours^28^. Our experimental approach to benchmark PA-ECM hydrogels against organoids and other in vitro platforms as models for PDAC is outlined in Fig. 1i.

**Fig. 2:**
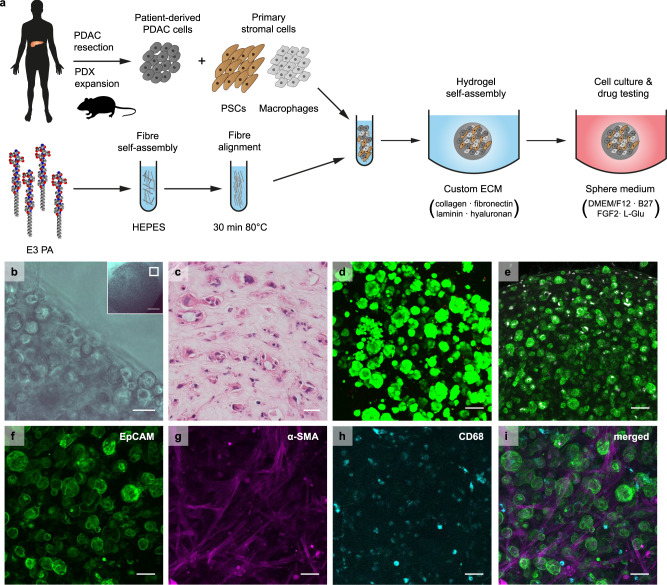
PA-ECM cultures for the ex vivo modelling of pancreatic cancer. **a** Schematic illustration of 3D cell culture in PA-ECM. **b** Brightfield micrograph of PDAC cells co-cultured with PSCs and macrophages in PA-ECM; insert indicates imaged area within a 5 µL hydrogel. Scale bar: 50 µm. Insert scale bar: 500 µm. **c** Haematoxylin (blue) and eosin (red) stain of PA-ECM hydrogel triple culture. Scale bar: 50 µm. **d** 3D projection of PDAC cells grown in PA-ECM for 14 days. Living cells were stained with calcein AM (green) and dead cells with ethidium homodimer (red). Scale bar: 100 µm. **e** 3D projection of PDAC cells co-cultured with PSCs and macrophages in PA-ECM hydrogels for 7 days and stained for EpCAM (green) and Ki-67 (white). Scale bar: 100 µm. **f**–**i** 3D projection of a PA-ECM hydrogel triple culture. PDAC cells were identified by EpCAM (green)(**f**), PSCs by α-SMA (magenta) (**g**) and macrophages by CD68 (cyan) (**h**) immunostaining. All cell types are shown on (**i**). Scale bars: 50 µm.

To validate the platform for cell culture, we generated 3D co-cultures of patient-derived primary PDAC cells and stromal cells, which we encapsulated in PA-ECM hydrogels (Fig. 2a). Cell encapsulation was verified by microscopy (Fig. 2b). Over time, PDAC cells formed duct-like colonies amid extensive stroma (Fig. 2c). After 14 days, these cultures remained highly viable, as indicated by live/dead staining (Fig. 2d), and proliferative, as evidenced by Ki-67 staining (Fig. 2e; Supplementary Fig. 1a, b). Average colony diameter after 7 days was consistent in PA and PA-ECM (58 µm) and, by day 21, it increased a further 10 µm in PA-only gels and 20 µm in PA-ECM (Supplementary Fig. 1c, d). Colony number and size were highly patient-specific and did not increase with higher ratios of stromal cells (Supplementary Fig. 1e, f). PDAC and stromal cells intermingled throughout the co-assembled matrices, maintaining their usual morphologies—elongated PSCs and rounded macrophages (Fig. 2f–i). In 2D, spheres and collagen gels, colonies were generally solid, without lumina. This contrasts with PA-ECM and organoid colonies, which were predominantly hollow or toroidal (Supplementary Fig. 1g, h), thereby preserving the topology of pancreatic ducts.

### Ex vivo models of PDAC are transcriptionally distinct

**Fig. 3:**
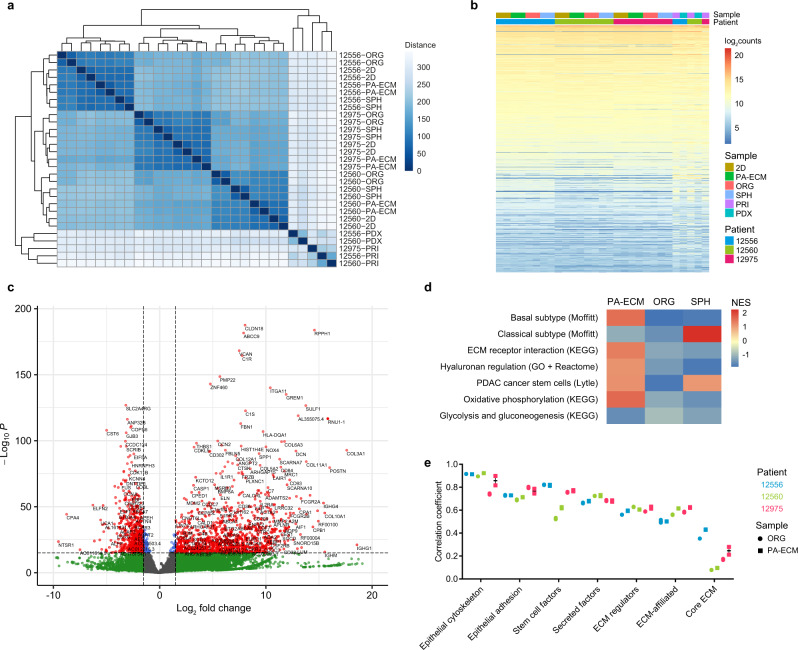
Transcriptomic analysis of ex vivo models of PDAC. **a** Hierarchically clustered distance matrix of the PDAC samples analysed by RNA sequencing. Distance is shown in colour-coded arbitrary units. **b** Regularised log_2_ expression of the top 20,000 genes expressed in all samples. **c** Volcano plot showing gene enrichment in primary tumours compared to PA-ECM monocultures, with correction for interpatient differences. **d** Heatmap depicting pathways identified by gene set enrichment analysis that are up- or downregulated in 3D models with respect to 2D monolayers. Normalised enrichment scores (NES) are colour-coded. **e** Correlation of epithelial and matrisome gene list expression between primary PDAC tissues and their corresponding organoids and PA-ECM cultures. Mean and range are shown for two biologically independent replicates per sample type. ORG organoids, SPH spheres, PRI primary tumours.

To explore the overall differences between PA-ECM cultures and current in vitro models of PDAC (2D monolayers, Matrigel-embedded organoids and floating spheres), we analysed monocultures of patient-derived cancer cells by RNA-seq and compared the results to their corresponding primary and PDX tumours. All ex vivo cultures clustered by patient (Fig. 3a), which indicates that patient-specific transcriptional programs were maintained. Although overall gene expression was similar in vivo and ex vivo (Fig. 3b), there was a notable distance between the tumour samples and the monocultures (Fig. 3a; Supplementary Fig. 2a). This may reflect gene expression from other cell types within the tumour microenvironment. Analysis of differential expression between PA-ECM cultures and primary tumours confirmed that this difference was largely due to the higher expression of interstitial matrix proteins and other stromal factors in the primary tissue samples (Fig. 3c). This was also observed when comparing organoids to primary samples (Supplementary Fig. 2b). Interestingly, differential expression analysis between the 3D models and 2D monolayers revealed enrichment in receptor-binding matrix components, as well as hyaluronan regulators, in PA-ECM hydrogels (Fig. 3d). Moreover, PA-ECM cultures favoured the basal (or squamous) PDAC subtype, which corresponded to the patients’ primary tumours analysed in this study (Supplementary Fig. 2c–d), while spheres were highly enriched in the classical (or pancreatic progenitor) subtype. Both PA-ECM cultures and spheres were highly enriched in the pancreatic CSC signature recently identified by Lytle et al.^29^ (Supplementary Data 1), while organoids showed negative enrichment, suggestive of a more differentiated phenotype (Fig. 3d; Supplementary Fig. 2e). PA-ECM cultures were also enriched in genes involved in oxidative phosphorylation, a hallmark of pancreatic CSCs^30^. Linear regression revealed that cancer cell-specific signatures, especially the CSC signature, were strongly correlated between primary tumours and their ex vivo models, while matrix components were only weakly correlated, especially the core ECM (Fig. 3e). Overall, 15 out of 21 correlations were stronger for PA-ECM cultures than for organoids. Interpatient differences in CSC marker expression were also reflected better in PA-ECM cultures than in other ex vivo models (Supplementary Fig. 2f). Taken together, these findings suggest that self-assembled PDAC cultures in PA-ECM maintain the transcriptional cancer (stem) cell signatures of the corresponding human tumours, while matrisome components are relatively underrepresented in the absence of stromal cells.

### PA-ECM cultures contain functional CSCs

**Fig. 4:**
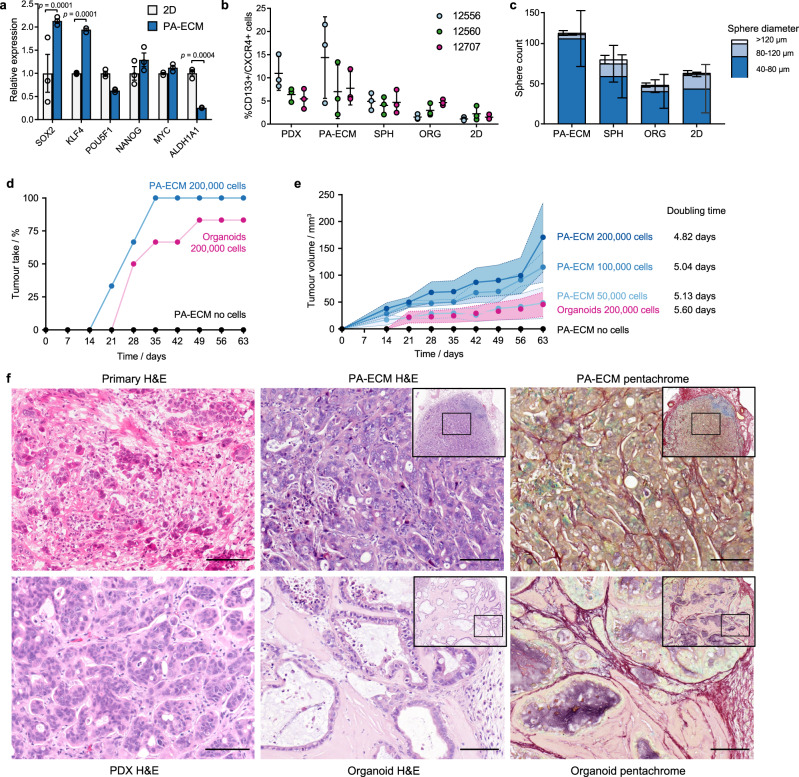
CSC enrichment in PA-ECM cultures. **a** Expression of CSC factors by PDAC cells (12560) cultured in PA-ECM compared to 2D monolayers (mean ± SD; *n* = 3 biological replicates). **b** Percentage of CD133 + /CXCR4 + PDAC cells in PDX and ex vivo PDAC models as assayed by flow cytometry in three matched experiments (mean ± SD). **c** Number of spheres by size range formed by PDAC cells (12560) derived from PA-ECM cultures, primary spheres, organoids and 2D monolayers (mean ± SD; *n* = 3 biological replicates). **d** Engraftment rate of PA-ECM and organoid PDAC cultures (12560) implanted in nude mice (three mice with two tumours per condition). **e** Volume of tumours grown in nude mice from implanted PA-ECM and organoid cultures carrying increasing numbers of PDAC cells (12560) (three mice with two tumours per condition). Shaded areas denote standard deviation around mean. **f** Representative histology images of PDAC tissues derived from patient 12560, including primary tumour, patient-derived xenografts and tumours grown in nude mice from implanted PA-ECM and organoid cultures. Scale bars: 100 µm.

To ascertain that there is a more appropriate representation of CSCs in PA-ECM cultures compared to other models, stemness marker expression was validated by qPCR and flow cytometry. Compared to 2D monolayers, there was a twofold increase in the expression of the stemness-related transcription factors *SOX2* and *KLF4* in PA-ECM cultures (Fig. 4a) and a much higher proportion of CD133 + /CXCR4 + cells than in spheres or organoids, which falls in the range of matched PDX tumours (Fig. 4b; Supplementary Fig. 3a). Functionally, the high CSC content of PA-ECM cultures translated into an increased rate of sphere formation compared to cells harvested from monolayer, organoid or primary sphere cultures (Fig. 4c). To confirm that these cultures maintained their tumorigenic potential, we implanted PA-ECM cultures and organoids subcutaneously into the flanks of nude mice. Implanted PA-ECM cultures resulted in a higher and accelerated engraftment rate compared to implanted organoids (Fig. 4d). Faster growth and larger tumour volumes were achieved by increasing the number of cells encapsulated in the implanted PA-ECM hydrogels (Fig. 4e). These implants grew comparatively faster than their corresponding PDX models (Supplementary Fig. 3b). For the same number of implanted cells, PA-ECM cultures resulted in tumours that were four times larger than those derived from organoids (Fig. 4e). The histological make-up of the tumours was also distinct. Those grown from PA-ECM implants were poorly differentiated like their primary tumour, with few discernible epithelial structures. In contrast, organoid-derived tumours were more well-differentiated and arranged into more organised duct-like epithelia (Fig. 4f; Supplementary Fig. 3c). Moreover, the matrix composition of the tumours was different, as shown by pentachrome staining: PA-ECM culture–derived tumours presented abundant collagen (shown in red) among the cancer cells, while in organoid-derived tumours collagen was localised to the stromal interface, mostly away from the cancer cells, and there were multiple mucinous cores (sulphated glycans in violet). These findings suggest that PDAC cells expanded as PA-ECM cultures and organoids differ significantly in their degree of differentiation, which is likely to impact interactions between the cancer cells and the surrounding stroma.

### Increased control over cellular phenotypes in PA-ECM hydrogels

**Fig. 5:**
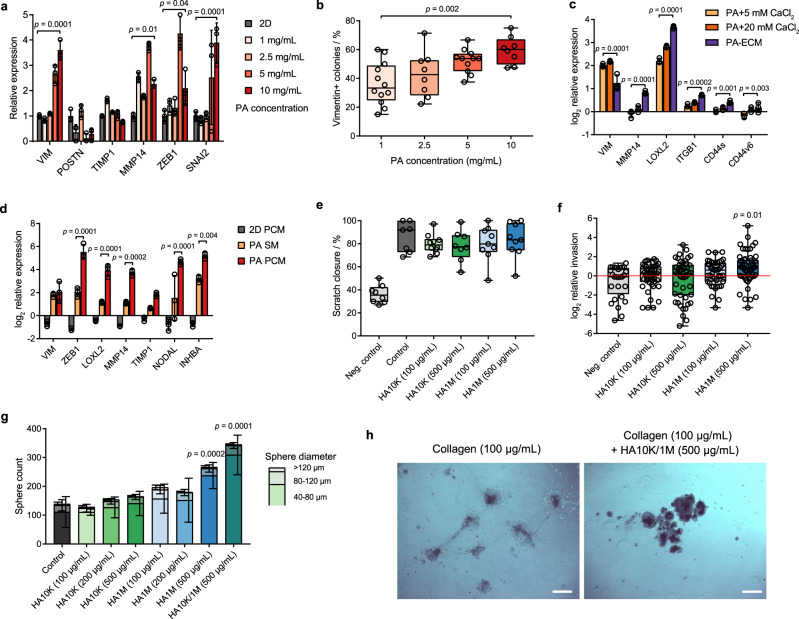
Control over PDAC cell phenotypes in PA hydrogels. **a** Gene expression of PDAC cells (12560) as a function of PA concentration relative to 2D controls (mean ± SD; *n* = 3 biological replicates). *p* values were calculated by two-way ANOVA with Bonferroni correction. **b** Percentage of vimentin-positive PDAC colonies (12560) as a function of PA concentration as quantified from immunofluorescence micrographs. Box plots indicate range, interquartile range and median (*n* = 8 biological replicates). *p* values were calculated by one-way ANOVA with Bonferroni correction. **c** Log_2_ normalised expression of PDAC cells (12560) grown in PA hydrogels assembled in different gelling conditions relative to the 2D baseline (mean ± SD; *n* = 3 biological replicates). *p* values were calculated by two-way ANOVA with Bonferroni correction. **d** Log_2_ normalised gene expression of PDAC cells (12560) cultured in PA hydrogels in sphere medium (SM) and PSC-conditioned medium (PCM) relative to the 2D SM baseline (mean ± SD; *n* = 3 biological replicates). *p* values were calculated by two-way ANOVA with Bonferroni correction. **e** Scratch closure of PDAC cells (12560) cells in the presence of hyaluronan. Box plots indicate range, interquartile range and median (*n* = 12 biological replicates in three experiments). **f** Log_2_ normalised invasion of PDAC cells (12560) across collagen-coated transwells as a function of the concentration of low (10 kDa) and high (1 MDa) molecular mass hyaluronan. Box plots indicate range, interquartile range and median (*n* = 42 biological replicates in 6 experiments). *p* values were calculated by one-way ANOVA with Bonferroni correction. **g** Number of spheres formed by PDAC cells (12560) as a function of the size and concentration of hyaluronan in the medium (mean ± SD; *n* = 6 biological replicates in three experiments). *p* values were calculated by two-way ANOVA with Bonferroni correction. **h** Brightfield images of PDAC cell colonies (12560) formed on low-attachment plates coated with collagen, with and without 10 kDa/1 MDa hyaluronan in the medium. Scale bars: 500 µm.

A key advantage of PA-ECM hydrogels is their tuneability, which enables separate control over their physical properties and composition, thereby facilitating the study of phenotypic responses to specific stimuli. To illustrate this, we investigated the cellular plasticity of PDAC cultures in PA-ECM hydrogels. Since cellular plasticity is one of the hallmarks of CSCs^31^, we hypothesised that cultures in self-assembling hydrogels would more readily acquire mesenchymal characteristics upon stimulation. To test this, we cultured PDAC cells in PA-only hydrogels of increasing fibre density as a strategy to mechanically promote EMT. This resulted in the upregulation of canonical EMT markers, such as vimentin and *MMP14*, as well as their upstream transcription factors *ZEB1* and *SNAI2* (Fig. 5a). At the protein level, the number of vimentin-positive colonies was increased (Fig. 5b), as was the total amount of vimentin (Supplementary Fig. 4a). These findings are in line with the observation that high epithelial vimentin correlates with densely packed fibrillar ECM in PDAC^32^. Increasing cell density had a similar effect (Supplementary Fig. 4b), which suggests analogous mechanotransduction. Unlike *MMP14*, other ECM regulators like periostin and *TIMP1* were downregulated in PDAC cells (Fig. 5a). In contrast, all three were upregulated in PSCs (Supplementary Fig. 4c, d), hinting at cell-lineage-specific differences in mechanotransduction.

Next, we hypothesised that modifying the composition of the gelling solution, as well as the medium, would enable control over the expression of niche-dependent genes. Indeed, PA-ECM co-assembled cultures displayed a clear upregulation of various ECM receptors (*ITGB1*, *CD44s*, *CD44v6*) and regulators (*MMP14*, *LOXL2*) compared to PA-only gels assembled in ECM-free CaCl_2_ solutions (Fig. 5c). Vimentin expression was increased in PA-only hydrogels, which can be rationalised by the crosslinking ability of CaCl_2_ resulting in the formation of more rigid hydrogels. We then used conditioned medium from primary PSCs as a model of paracrine stimulation and also observed an upregulation of genes involved in ECM remodelling and EMT (Fig. 5d), which was not observed in spheres or organoids (Supplementary Fig. 4e). On the other hand, conditioned medium from PDAC cells upregulated the expression of collagens in self-assembled PSC monocultures, enhancing the effects observed in 2D (Supplementary Fig. 4f). We also investigated macrophage cultures, whose gene expression upon polarisation with CSF1 was found to resemble that of tumour-associated macrophages from PDAC tumours (Supplementary Fig. 5a). Macrophages thrived as self-assembled monocultures (Supplementary Fig. 5b) and, upon exposure to both the PDAC and PSC-derived conditioned media, they exhibited a modified cytokine profile, particularly by upregulating IL6 (Supplementary Fig. 5c), which was validated by ELISA (Supplementary Fig. 5d). These paracrine effects may explain the relative proximity of macrophages to PDAC colonies in co-cultures in PA-ECM (Supplementary Fig. 5e).

We next specifically investigated the ECM polysaccharide hyaluronan as a proven critical component of PDAC^33^. Hyaluronan is highly abundant in the PDAC stroma (Supplementary Fig. 5f) and is known to upregulate key CSC- and EMT-related genes, such as *ZEB1*^34^ and *NANOG*^35^. PA-ECM cultures with hyaluronan revealed that these previously reported transcriptional effects are size- and concentration-dependent (Supplementary Fig. 5g). Functionally, hyaluronan did not stimulate migration in 2D (Fig. 5e), but it did so in 3D in a size- and concentration-dependent manner (Fig. 5f; Supplementary Fig. 5h) and also enhanced sphere formation (Fig. 5g). Strikingly, the combination of both low- and high-molecular-mass hyaluronan resulted in the highest increase in sphere formation, which supports the previously proposed notion of synergistic effects between both forms of hyaluronan on PDAC cells^36^. Such combination was able to rescue sphere formation in PDAC cells seeded in suspension on collagen-coated plates, which otherwise form semi-adherent monolayers (Fig. 5h). These effects could be mediated by CD44, which is one of the most widely expressed hyaluronan receptors in PDAC and a known CSC marker, although other receptors such as HMMR could also play a role. These findings highlight the regulatory functions that hyaluronan exerts on PDAC cells and how these can be dissected in PA-ECM hydrogels according to features such as polymer size and concentration.

### Multicellular PA-ECM cultures enhance PDAC matrisome recapitulation

Despite the inclusion of hyaluronan, collagen, laminin and fibronectin in our custom ECM, many core matrix proteins remained underrepresented in PA-ECM monocultures, as shown in Fig. 3c. In PDAC, the primary source of ECM is the stromal compartment, which, instructed by the PDAC cells, produces large quantities of diverse matrix proteins^24^. Thus, to enhance the ex vivo recapitulation of the stroma of our platform, we created multicellular PA-ECM cultures by also incorporating primary PSCs and macrophages (triple cultures), as described above (Fig. 2a). Preliminary analysis by ELISA showed that both PA-ECM and organoid cultures secreted a variety of cytokines, of which IL-6 and CCL2 were the most abundant in triple cultures compared to monocultures (Supplementary Fig. 5d). Matrix deposition by multicellular PA-ECM cultures and organoids was quantified by mass spectrometry and compared to that of corresponding primary and PDX tumours. The abundance and composition of the matrisome of the primary tumours was considerably heterogeneous, which was also reflected by both the PDX and ex vivo models (Supplementary Data 2). In particular, the least differentiated tumours (12560 and 12707) presented the most abundant matrisomes both in vivo and ex vivo (Supplementary Fig. 6a, b; Supplementary Data 2). For all primary tumours, collagens were the most abundant proteins (Supplementary Fig. 6c), especially collagen type I (Supplementary Fig. 6d), while glycoproteins and proteoglycans were less abundant and had a more variable distribution (Supplementary Fig. 6e, f), which was validated by immunohistochemistry (Supplementary Fig. 7).

**Fig. 6:**
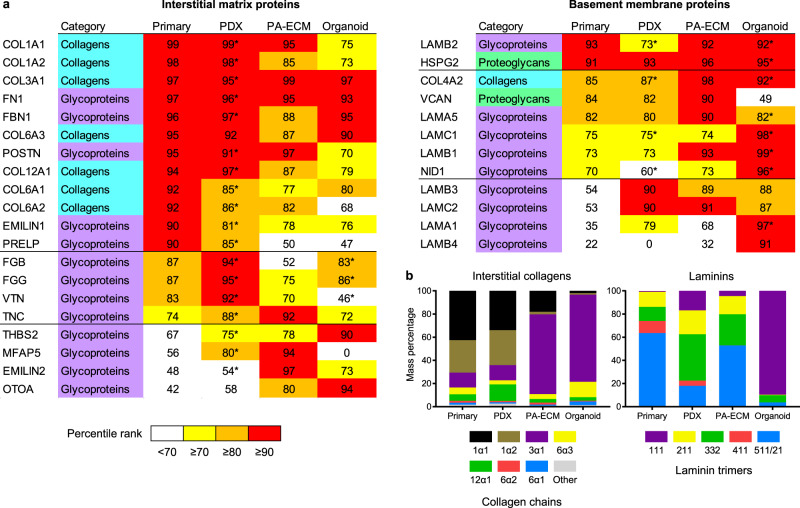
Core matrisome recapitulation in PDX, PA-ECM and organoid models. **a** Heatmap of the top matrisome proteins detected which fall on or above the 90th percentile rank in abundance (mean across all patients) in at least one of the sample categories. For the PDX and Matrigel samples, abundance was calculated as the sum of the human and murine peptides. Those proteins for which >50% of the peptides detected were of murine origin are indicated with an asterisk. **b** Distribution of interstitial collagen chains and laminin trimers for all sample categories. Data correspond to the mean across all patients.

Comparison between the top matrisome proteins from in vivo and ex vivo samples revealed a rich basement membrane in both PA-ECM cultures and organoids and a moderate underrepresentation of interstitial matrix proteins ex vivo (Fig. 6a). This highlights the inherent limitations of short-term cultures for the modelling of fibrotic tissues, since the bulk of interstitial matrix deposition and remodelling occurs as a long-term process complemented by the daily turnover of a much smaller sacrificial ECM pool^37^. This notion is further supported by the observation that ex vivo models are richer in collagen type III than type I (Fig. 6b), which is characteristic of early ECM deposition during wound healing and fibrosis. Despite this limitation, PA-ECM cultures exhibited very high fibrillar collagen abundance, while Matrigel-embedded organoids were comparatively deficient in collagen type I, the most abundant matrisome protein in all primary tumour samples. As for the main proteins of the basement membrane, laminins, PA-ECM cultures presented a very similar composition to primary tissues, while organoids differed significantly (Fig. 6b). For example, laminin 111, a key constituent of Matrigel, makes up 90% of the laminins in organoid cultures, but less than 1% in primary tissues. This is due to the fact that, in humans, laminin 111 is not expressed postnatally in most tissues, including the pancreas^38^, and therefore does not constitute a representative ECM protein for the modelling of PDAC and other tissues.

**Fig. 7:**
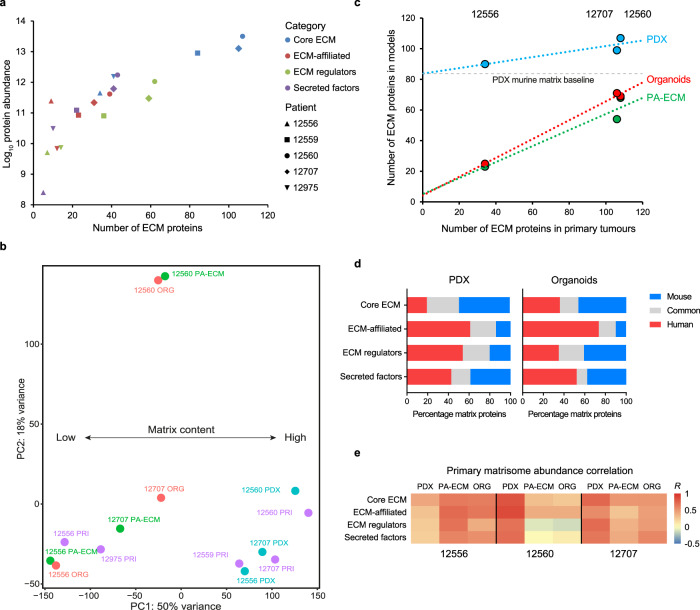
In vivo and ex vivo PDAC matrisome analysis. **a** Scatter plot showing the total abundance and number of proteins of each matrisome category for each primary tumour analysed. **b** Principal component analysis of the core matrisome datasets from patients 12556, 12559, 12560, 12707 and 12975. **c** Scatter plot showing the correlation between the number of proteins of the primary core matrisomes and those of their corresponding PDX, PA-ECM and organoid models. The PDX murine baseline indicates the number of murine ECM proteins predicted to reside at the site of PDX implantation. **d** Percentage of matrisome peptides detected in the PDX and organoid samples which are of human, mouse or common origin. Common peptides are identical in both species. **e** Colour-coded correlation between the core matrisome protein abundances in primary tumours and their corresponding PDX, PA-ECM and organoid models. *R* is the correlation coefficient.

These observations are in agreement with recently published data^39^ and expand on those findings by showing that patients with highly abundant ECMs also harbour the most diverse matrisomes (Fig. 7a). In consequence, we hypothesised that patients would cluster according to their overall matrisome content and this was indeed the case (Fig. 7b). When analysing the total number of detected proteins by linear regression, both the PA-ECM cultures and organoids were predictive of overall ECM content, while PDX models were less reliable due to the large number of murine ECM proteins (Fig. 7c). Further analysis of the origin of matrisome proteins from the PDX and organoid models revealed that over 50% of core ECM proteins were of mouse origin (Fig. 7d). These were mostly interstitial ECM proteins such as fibronectin, fibrinogen and periostin for PDX tumours, whereas most murine proteins in organoids were basement membrane constituents of Matrigel. Due to this overrepresentation of basement membrane proteins in organoids, the core ECM abundance correlations with the corresponding primary tumours were much weaker than those observed for PA-ECM cultures (Fig. 7e; Supplementary Fig. 8). These differences in correlation with patient-specific matrisomes are in line with the transcriptomic data shown in Fig. 3e, i.e., primary tumours with low ECM content (12556) show stronger correlation with the respective ex vivo models than those primary tumours with high ECM content (12560). The stronger correlations observed between the primary tumours and the PA-ECM models compared to the corresponding organoids are also found in both transcriptomic and proteomic datasets, which clearly indicates that PA-ECM cultures enhance matrisome recapitulation.

### In vivo drug responses are reproduced in self-assembled PDAC cultures

**Fig. 8:**
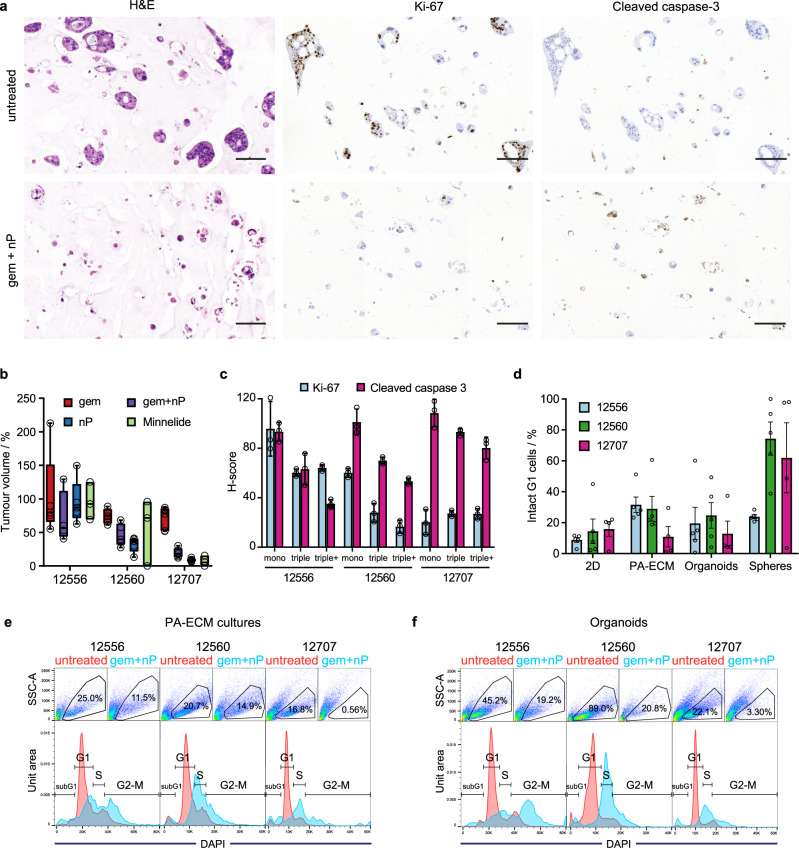
PDAC treatment response in PA-ECM and organoid cultures. **a** Histology of PDAC (12560) mono- and triple cultures in PA-ECM treated with gemcitabine/nab-paclitaxel. Cells were stained with H&E to assess cellularity, Ki-67 for proliferation and cleaved caspase-3 for apoptosis. Scale bars: 100 µm. **b** In vivo response of PDX tumours to chemotherapy depicted as percentage of tumour volume post-treatment relative to controls (0.9% NaCl). Box plots indicate range, interquartile range and median. For each patient and condition, *n* = 5 mice with one tumour each. **c** Quantification of Ki-67 and cleaved caspase-3 staining of PDAC mono- and triple cultures in PA-ECM treated with gemcitabine/nab-paclitaxel. Triple+ cultures contained twice the number of PSCs (mean ± SD; *n* = 3 biological replicates). **d** Response of PA-ECM and organoid cultures to gemcitabine/nab-paclitaxel as assayed by flow cytometry. Percentages represent the proportion of cells that were both intact and in G1 phase compared to untreated controls (mean ± SD; *n* = 5 biological replicates). **e**, **f** Flow cytometry plots showing the cell cycle arrest of PA-ECM (**e**) and organoid (**f**) cultures treated with gemcitabine/nab-paclitaxel. The upper panels show the gated populations of intact cells. The lower panels depict the cell cycle profiles of untreated (red) and treated (blue) samples gated to exclude debris and doublets.

Since both CSCs and the abundant matrix are known to contribute to therapy resistance in PDAC^24,40^, and PA-ECM cultures recapitulate these features more closely than organoids, we hypothesised that they should also better reflect in vivo drug response. PA-ECM cultures are amenable to analysis of drug response by histology, which enables the quantification of cellularity, and by immunohistochemistry, which enables the quantification of proliferative and apoptotic cells by staining for Ki-67 and cleaved caspase-3, respectively (Fig. 8a). In order to benchmark ex vivo drug responses, we used in vivo responses of PDX tumours to gemcitabine/nab-paclitaxel (a standard-of-care treatment for PDAC) as a reference. We selected patients whose PDX models exhibited characteristically low, moderate and high sensitivity to chemotherapy (Fig. 8b). We then quantified the response of the corresponding PDAC mono- and multicellular cultures in PA-ECM hydrogels to gemcitabine/nab-paclitaxel (Fig. 8c). As expected, the highest rate of apoptosis and lowest proliferation was observed in the most chemosensitive patient, 12707, while the opposite was observed for the most resistant patient, 12556. The patient exhibiting moderate chemosensitivity, 12560, also showed an intermediate response ex vivo.

Interestingly, the inclusion of PSCs and macrophages in these cultures resulted in a decrease in the rate of apoptosis, but did not alter cell proliferation, which suggests that stromal cells promote cell-intrinsic chemoresistance, as previously reported^41^. When we doubled the number of incorporated PSCs, there was a proportional decrease in the number of apoptotic cells, which further supports this notion. Moreover, qualitative analysis of responses to a variety of chemotherapeutic agents showed a larger number of resistant colonies in multicellular cultures compared to monocultures (Supplementary Fig. 9a). This difference could even be observed for cultures treated with the potent diterpenoid triptolide, whose prodrug minnelide is currently in clinical testing (NCT03117920). An alternative method for assessing PDAC drug response in PA-ECM is the quantification of the tetrasaccharide CA19-9, which is used as a circulating biomarker to track tumour burden and drug response in PDAC patients^42^. However, we found no correlation between secreted CA19-9 and response to treatment for any of our tested ex vivo models (Supplementary Fig. 9b). Of note, one of the analysed tumours did not produce any CA19-9, a condition known as Lewis-negative, which further limits the relevance of this method.

Instead, to more accurately and mechanistically quantify treatment responses, we assayed cell cycle profiles in various ex vivo conditions. This should provide early evidence of response to both the gemcitabine and nab-paclitaxel, as these result in cell cycle arrest in M and G2 phase, respectively. Thus, the number of viable cells remaining in G1 upon treatment should constitute a suitable surrogate marker for drug response and therefore correlate with the in vivo PDX tumour volume following treatment. Intriguingly, only PA-ECM cultures consistently reproduced the in vivo response, while spheres and 2D monolayers actually showed the opposite response pattern (Fig. 8d). For organoids, response was variable and did not correlate with the in vivo data. Examination of the cell cycle profiles of ex vivo cultures revealed enhanced resistance in PA-ECM gels, denoted by a higher proportion of treated cells in G1 for all tested tumours (Fig. 8e–f). When PSCs were incorporated into these cultures, they remained present in higher numbers in PA-ECM gels than in 2D or organoids after 10 days in culture (Supplementary Fig. 9c), which suggests that PA-ECM hydrogels enhanced the proliferation and/or viability of stromal cells. Upon treatment with gemcitabine/nab-paclitaxel, PSCs showed a further three-fold enrichment in all conditions, which indicates resistance to cell cycle arrest.

However, when cultures were treated with triptolide, both PSCs and PDAC cells were eradicated. PDAC monocultures treated with triptolide showed large differences in response between the resistant and sensitive patients, with both the PA-ECM and organoids most closely reproducing the in vivo response (Supplementary Fig. 9d). Interestingly, while response to gemcitabine/nab-paclitaxel seemed to correlate with the proportion of CSCs in culture, response to triptolide did not correlate with the presence of CSCs (Supplementary Fig. 9e). These data are not in line with the concept that triptolide specifically targets CSCs^43^, which was proposed based on studies using immortalised cell lines. Instead our results suggest that triptolide efficiently targets both the CSC and non-CSC. Overall, our findings indicate that primary PA-ECM cultures mimic in vivo drug responses and may constitute a useful platform for ex vivo drug testing and to investigate the specific dependencies and mechanisms of resistant cells.

## Discussion

Over the past decade, increasing interest in the ex vivo recapitulation of in vivo tumour biology and stem cell niches has spurred the development of organoid cultures to model malignancies such as PDAC^44^. Although organoids constitute a very useful model of ductal differentiation, PDAC cells in such cultures are dependent on a diverse cocktail of factors originally identified in the intestinal crypt, such as R-spondin and noggin, with unclear relevance for PDAC. These factors promote the self-renewal of cells expressing LGR5^45^, but CSCs in PDAC do not seem to express LGR5^46^. This may explain, at least in part, our finding that CSC content in organoids was significantly lower than in PA-ECM cultures and spheres. On the other hand, self-assembling hydrogels have been widely used to recreate normal stem cell niches for the modelling of neurogenesis^47^, osteogenesis^48^ and chondrogenesis^49^, often incorporating ECM molecules such as heparin^50^ and hyaluronan^51^. Here we show that more comprehensive PA-ECM hydrogels can be employed to mimic key features of the PDAC microenvironment in a defined manner, resulting in cultures that are enriched in highly tumourigenic CSCs. As proof of concept, we show that the expression of EMT markers and functional responses, such as 3D migration and sphere formation can be modulated by controlling specific properties of the hydrogels, such as fibre density or the size and concentration of hyaluronan, which has long been suspected of promoting cancer stemness^33^. In addition, the co-assembled PA-ECM hydrogels provide a fibrillar microenvironment with tuneable alignment. Such anisotropic arrangement of ECM fibres is commonly observed in PDAC tumours and appears to correlate with poor prognosis^32^.

We expect that future studies will shed more light on the exact composition of the pancreatic CSC niche, which remains unknown despite several promising studies^31,52,53^. Large-scale transcriptomic analysis of PDAC primary tumours has shown that laminin-332 has the highest hazard ratio among all matrisome proteins^39^. Notably, this matrix protein was highly abundant in PA-ECM cultures, whereas organoids are dominated by laminin-111, which is rarely found in primary tumours. Moreover, while the core matrisome of PA-ECM cultures reflected the fibrotic status of the corresponding primary tumours, organoid cultures contained very little collagen type I, despite it being the main component of the PDAC matrisome. Thus, the skewed ECM composition and the inadequate softness of Matrigel hampered a sufficient recapitulation of the PDAC matrisome, which only recently was shown to correlate with progression^39^ and poor prognosis^54^. Furthermore, the defined composition of PA-ECM hydrogels and the use of primary stromal cells instead of immortalised cell lines such as PS1 and THP-1 improve upon the design of previous studies^55^ and enhance the clinical adequacy of the platform. In the context of in vivo studies, PA-ECM hydrogels appear to be a suitable carrier for tumour implantation and may be considered as an alternative to Matrigel. Methodological improvements such as orthotopic implantation and the use of humanised mice could further strengthen its use, especially in translational studies.

The interplay between niche-dependent phenotypes, such as stemness, EMT, fibroblast activation and ECM deposition has been recognised as a critical factor in PDAC chemoresistance^24^. Accordingly, comprehensive bottom-up approaches like ours are necessary to accurately reflect clinical responses^56^. Indeed, drug sensitivity for established cell lines and traditional 2D cultures rarely correlates with clinical responses, while 3D hydrogel cultures often exhibit more authentic responses^57–59^. Consistently, our data show that neither 2D cultures nor spheres grown in suspension were able to accurately reflect the in vivo response of PDX models. On the other hand, PA-ECM cultures faithfully reproduced the in vivo response when assessed as cytotoxicity and cell cycle arrest. Overall, organoids exhibited a weaker correlation, which may be related to their reduced CSC content and insufficient recapitulation of the PDAC matrisome. Although our data suggest that PA-ECM hydrogels represent a useful platform for drug testing using patient-derived cells, studies with larger cohorts and a wider distribution of tumour stage, site and sampling technique (resection, solid and liquid biopsy) will be required to fully establish this platform in the preclinical arena. More exhaustive studies should also determine whether optimisation of our tuneable self-assembling approach is necessary to more closely match certain biological features such as tumour grade and subtype. Whereas such optimisation is generally not possible with substrates like Matrigel due to their reduced tractability, it is allowed by the modular nature of our system, including the customisation of the ECM, cellular ratios and medium composition, as well as the physical properties of the co-assembled hydrogels. However, we hypothesise that major adaptations of the system may not be needed given the observed maintenance of patient-specific transcriptomic, proteomic and drug response signatures.

Another important aspect to consider for preclinical implementation is scalability, which is also facilitated by the wide range of hydrogel volume (1–100 µL) and peptide concentration (1–20 mg/mL) at which this platform can operate. Lower volumes might be particularly useful when working with (liquid) biopsies from non-resectable stage III and IV patients due to the reduced number of cells that are typically obtained. Such low volumes also present the added benefit of being more affordable, a critical consideration for clinical implementation. Consistency and reproducibility of the gels at different volumes and for different co-assembling matrices should also be confirmed to ensure reliable results in any potential translational applications. More widespread availability and GMP certification of commercially available peptides should also help establish their consistency at the preclinical stage. Preliminary studies combining self-assembly with biofabrication^22^ and microfluidics^60^ open the door to the development of more advanced ex vivo models capable of recreating, for example, vascular and neuroendocrine components, which may further enhance the recapitulation of each patient’s characteristic drug resistance. In particular, these approaches may enable the modelling of cell-extrinsic resistance mechanisms such as hypoxia and interstitial fluid pressure, which are critical factors in PDAC drug response^24^. By linking various co-assembled matrices representative of different tissues, such approaches may also enable the study of metastatic dissemination and homing. Control over the molecular and physical properties of the cultures over time may also enhance the evaluation of functional endpoints, such as invasion and stemness^61^.

In the future, we hope to obtain a more thorough understanding of the cancer cell populations in self-assembled cultures as compared to their corresponding tumours (e.g., by single-cell and spatial transcriptomics), as well as their metabolic status, which should enable us to refine the system further and expand our systematic comparison with models such as spheres and organoids. Recently, in vivo experimentation has demonstrated that the transcriptomic profile and progression of PDAC is conditioned by the microenvironment^62^; reproducing these findings ex vivo would also be of great value given the marked heterogeneity of PDAC tumours. Ultimately, the major challenge remaining is to establish the fidelity of the system across a wider cohort of patients, especially in terms of drug resistance and sensitivity. To ensure consistency, clinical and preclinical testing with patients and patient-derived PA-ECM cultures should be run in parallel as co-clinical trials^63^. Although, so far, such an approach has been explored in mouse models only, it may become a new standard for the validation of comprehensively characterised ex vivo platforms like the one presented here.

## Methods

### Primary human tissue

Patient samples were collected through the ARC-Net Biobank of the University and Hospital Trust of Verona approved by the Verona University Hospital Ethics Committee (Program 1885 protocol 52,438 23/11/2010, program 2172 protocol 26,773 23/05/2012; Supplementary Table 2). Informed consent was obtained from all participants. Patient-derived xenografts (PDXs) were produced under decree no. 107/2012-B and 108/2012-B by the Italian Ministry of Health based on the legislative decree 106/92 regarding the protection of animals used in scientific research. PDX tissues, grown subcutaneously in the flank of immunodeficient NMRI:*Foxn1*^*nu/nu*^ mice (Charles River, UK), were used to isolate primary PDAC cells with 2% collagenase P (Roche, UK) and 1 mg/mL dispase (Life Technologies, UK). Mice were housed in a dedicated facility at 24 °C with a 12-h light/dark cycle and 50% humidity. Cells were grown in RPMI medium (Life Technologies, UK) supplemented with 10% foetal bovine serum at 37 °C and 5% CO_2_. For experiments, cells were maintained in sphere medium: DMEM/F12 supplemented with 2% B27 (Life Technologies, UK), 20 ng/mL FGF2 (PeproTech, UK) and 2 mM L-glutamine (Life Technologies, UK), while organoids were grown in organoid medium^6^. For multicellular cultures, PDAC cells were co-cultured with primary PSCs isolated from PDAC patient-derived tissues collected at the Technical University of Munich and approved by the Faculty of Medicine Ethics Committee (ethical approval 5510/12), and with primary macrophages. These were derived from circulating monocytes obtained from the blood of healthy donors (REC reference 17/EE/0182) and polarised with CSF1 (20 ng/mL) for 5 days.

### PA design and synthesis

E3 PA (C_16_-V_3_A_3_E_3_) was chosen due to its robust and tuneable physical properties^27^, as well as its ability to co-assemble with ECM molecules through electrostatic interactions (Fig. 1a) and to enable co-assembled nanofibre alignment^24^. Other negatively-charged PAs exhibited weaker assembly, while positively-charged PAs, such as K3 PA (C_16_-V_3_A_3_K_3_) were rejected due to their ability to disrupt cell membranes^64^. E3 PA was produced by solid-phase peptide synthesis as previously reported^65^. Further information is given in the supplementary methods.

### Cell culture in PA-ECM

E3 PA was dissolved in HEPES buffer (10 mM HEPES, 3 mM KCl, 150 mM NaCl) at pH 7.4 to a concentration of 10 mg/mL, transiently heated to 80 °C for 30 min and mixed with cells to obtain hydrogels (2.5 × 104 cells/cell type/5 µL hydrogel) as illustrated in Fig. 2a. For the assembly of the hydrogels, a custom ECM solution was prepared by diluting collagen type I (Advanced BioMatrix, USA) tenfold in HEPES buffer to a final concentration of 500 μg/mL, followed by dilutions of fibronectin (50 μg/mL final concentration) (R&D Systems, USA), laminin (50 μg/mL) (R&D Systems, USA), 10 kDa hyaluronan (500 μg/mL; Lifecore, UK) and 1 MDa hyaluronan (500 μg/mL; Lifecore, UK). These ECM macromolecules, in similar ratios (Supplementary Fig. 10), are known to be among the most abundant in the PDAC ECM^39^. In 96-well plates, 5 μL PA cultures were co-assembled with 50 μL of ECM. Alternatively, when evaluating the effects of paracrine factors or physical parameters on PA cultures, 20 μL of 20 mM CaCl_2_ were used as gelling solution to avoid the confounding biological effects of the custom ECM components.

### Characterisation of PA-ECM cultures

Cell viability was assessed using a live/dead kit (Life Technologies, UK) and imaged on a Zeiss 710 confocal microscope. For immunofluorescence imaging, samples were fixed in 4% paraformaldehyde, permeabilized with 0.25% Triton X-100 and blocked with 5% bovine serum albumin prior to antibody incubation (Supplementary Table 3). Z-stack images were taken at 10 µm intervals over a distance of 250–350 µm and analysed on ImageJ 1.52 u (NIH, USA). For gene expression analysis, total RNA was extracted with TRI Reagent (Sigma-Aldrich, UK) and reverse-transcribed into cDNA using the QuantiTect Reverse Transcription Kit (Qiagen, UK). qPCR was performed using the PerfeCTa SYBR Green FastMix Low Rox (Quanta Biosciences, UK) and gene-specific primers (Supplementary Table 4) on a QuantStudio 7 system (Applied Biosystems, UK). Results were analysed with the comparative C_t_ method and normalised to the expression of the housekeeping gene *RPS13*. Selected genes were validated at the protein level by Western blot. Briefly, cell lysates were prepared in RIPA buffer, separated by SDS-PAGE and blotted onto nitrocellulose membranes. After blocking with 5% bovine serum albumin, primary antibodies (Supplementary Table 3) were incubated overnight, followed by HRP-linked secondary antibodies and ECL Prime Western Blot Detection Reagent (GE Healthcare, USA). Chemiluminescence was detected on an Amersham Imager 600 (GE Healthcare, USA).

### Functional assays

The sphere-forming capacity of PDAC cells was evaluated following established methods^53^ at a density of 1 × 10^3^ cells/mL and quantified on a CASY TTC counter (Innovatis, Switzerland). Migration was assessed by measuring the scratch closure of monolayers cultured in 24-well plates (2 × 10^5^ cells/well) after exposure to hyaluronan for 20 h. Invasion was assayed in 0.8 µm pore-sized transwell inserts (Life Technologies, UK) coated with 70 µL of collagen type I (0.1 mg/mL). 5 × 10^4^ cells were seeded per well and exposed to hyaluronan from the lower chamber. After 24 h, cells were fixed, stained with DAPI and imaged. Controls were run without hyaluronan and negative controls without foetal bovine serum.

### Transcriptomic analysis

PDAC cells were cultured for 7 days as 2D monolayers, PA-ECM cultures, Matrigel-embedded organoids and sphere cultures and RNA was extracted with the RNeasy mini kit (Qiagen, USA). Corresponding primary and PDX tissue samples were dissociated prior to RNA extraction. mRNA was enriched by polyA selection and transcriptomic analysis was performed on a NovaSeq 6000 (Illumina, USA). FASTQ files were checked for quality with FastQC and sortmerna was used to deplete rRNA from tissue-derived sequences. All sequences were then mapped to the primary assembly of GENCODE 30 using kallisto v0.45.0. Differential gene expression was analysed with DESeq2 and gene set enrichment with GSEA 4.0.1.

### Matrisome analysis

Matrix-enriched pellets from primary tumour, PDX, PA-ECM and organoid samples were generated using the CNMCS compartmental protein extraction kit (BioChain, US) and processed for mass spectrometry as previously described^66^. Further details are given in the supplementary methods. Peptides were detected on an LTQ-Orbitrap XL mass spectrometer (Thermo Scientific, USA). For protein identification, Mascot searches were run against the human Swiss-Prot database (PDX and organoid samples were also searched against the murine dataset), while PESCAL was used to calculate relative protein abundances (Supplementary Data 2).

### In vivo implantation of cell-laden hydrogels

PDAC cells (5 × 10^4^ – 2 × 10^5^) were cultured for 7 days in PA-ECM and organoid conditions, respectively, and then subcutaneously implanted into the flanks of female immunodeficient NMRI:*Foxn1*^*nu/nu*^ mice (*n* = 6/per group). Cell-free PA-ECM served as control. Tumour dimensions were measured with a calliper and volume was calculated using the formula (width^2^ × length)/2, where length is the longest dimension. After 9 weeks, when tumours reached a palpable size (>250–300 mm^3^), necropsy was performed, and tissues were processed for histology and immunohistochemistry. Procedures were conducted in accordance with institutional and national regulations (Animals in Science Regulation Unit, Home Office Science, London, UK; project license PPL 70/8129).

### In vitro drug testing

PDAC mono- and multicellular cultures were grown in PA-ECM, organoid, sphere and 2D adherent conditions for 7 days and then treated with gemcitabine (100 ng/mL) and nab-paclitaxel (10 µM), alone or in combination, as well as triptolide (25 nM), for another 3 days (flow cytometry) or 7 days (IHC).

### Flow cytometry

Cells were extracted by dissociating PA-ECM hydrogels, organoids, spheres and 2D monolayers in TrypLE Express (Gibco, USA) with a micropipette. Samples were normalised to 10^6^ cells/mL, blocked with Flebogamma (Grifols, Spain) and incubated with primary antibodies (Supplementary Table 3). For cell cycle analysis, cells were fixed with 4% paraformaldehyde, permeabilized with 0.25% Triton X-100 and stained with DAPI (10 µg/mL). Cells were analysed on an LSR Fortessa cell analyser (BD Biosciences, USA) and gated to exclude debris and doublets (Supplementary Fig. 11).

### Histology and immunohistochemistry

Primary tumour, PDX and PA-ECM samples were fixed in 4% paraformaldehyde and embedded in paraffin (hydrogel replicates were combined into one agarose gel prior to embedding). Samples were sectioned (5 µm), mounted onto glass slides, deparaffinized with xylene and rehydrated. Antigen retrieval was performed at 95 °C in sodium citrate buffer. Sample morphology was examined by Mayer’s haematoxylin and eosin staining. Proteins were detected using primary antibodies (Supplementary Table 3), followed by HRP-conjugated secondary antibodies (Dako, USA), diaminobenzidine and Mayer’s haematoxylin counterstaining. Hyaluronan was stained with biotin-conjugated HABP2 and specificity was confirmed with hyaluronidase (50 units; Sigma-Aldrich, US) as previously reported^67^. Pentachrome staining was performed as previously reported^68^. Samples were imaged on a Pannoramic 250 Flash III scanner (3DHISTECH, Hungary). To evaluate drug response, the intensity of staining was automatically scored into four categories (negative, weak, moderate, strong) and calculated thus: H-score = [1 × (% area “weak”) + 2 × (% area “moderate”) + 3 × (% area “strong”)].

### Statistics and reproducibility

Data are shown as mean ± standard deviation, unless otherwise specified. Multiple comparisons were performed by one-way or two-way ANOVA with Bonferroni correction in Prism 7.05 (GraphPad, USA); the significance threshold was set at *p* ≤ 0.05 (Supplementary Data 3). Heatmaps, principal component analysis and linear regression plots were generated in R 3.5.3 (R Foundation, USA). The electron micrographs in Fig. 2b–e are representative of three independently synthesised hydrogels per condition. The micrographs in Fig. 2b–i are representative of over 100 cell-laden hydrogels prepared over a 4-year period. The histology images in Fig. 4f and Supplementary Fig. S3c are representative of three independent in vivo experiments. The brightfield micrographs in Fig. 5h are representative of three independent experiments using cells from two different patients. The ex vivo histology images in Fig. 8a are representative of three independent experiments using cells from three different patients. The H&E images in Supplementary Fig. 1d are representative of four individual gels per timepoint and condition. The confocal images in Supplementary Fig. 1e are representative of 16 individual gels. The brightfield images in Supplementary Fig. 1h are representative of at least 10 samples per condition. The confocal images in Supplementary Fig. 4d are representative of three samples per condition. The confocal image in Supplementary Fig. 5b is representative of three independent experiments. The IHC image in Supplementary Fig. 5e is representative of co-cultures with samples from three different patients. The histology images in Supplementary Fig. 5f are representative of tumours from four different patients. The epifluorescence images in Supplementary Fig. 5h are representative of five independent experiments. The histology images in Supplementary Fig. 6a and Supplementary Fig. 7 are representative areas from 5 × 5 mm tumour pieces. The H&E images in Supplementary Fig. 9a are representative of five individual experiments with cells from five different patients.

### Reporting summary

Further information on research design is available in the Nature Research Reporting Summary linked to this article.

## Supplementary information

Supplementary Information

Description of Additional Supplementary Files

Supplementary Data 1

Supplementary Data 2

Supplementary Data 3

Reporting Summary

## Data Availability

Raw and processed transcriptomic data are available at the GEO database under accession number GSE139184. Proteomic data are available at the PRIDE database under accession number PXD013254. All data that support the findings of this study are available within the Article, Supplementary information and Source data file. Source data are provided with this paper.

## Source data

Source Data
