# Supplementary material for: Bioengineered 3D models of human pancreatic cancer recapitulate in vivo tumour biology
Source: Nat Commun. 2021 Sep 24;12:5623. doi: 10.1038/s41467-021-25921-9 (PMC8463670; doi:10.1038/s41467-021-25921-9)
Supplement: Supplementary file 1 — Supplementary Information [file 41467_2021_25921_MOESM1_ESM.pdf]

Bioengineered 3D models of human pancreatic cancer recapitulate *in vivo* tumour biology

David Osuna de la Peña, Sara Maria David Trabulo, Estelle Collin, Ying Liu, Shreya Sharma, Marianthi Tatari, Diana Behrens, Bruno Sainz, Mert Erkan, Rita T. Lawlor, Aldo Scarpa, Christopher Heeschen, Álvaro Mata, Daniela Loessner

Supplementary Information

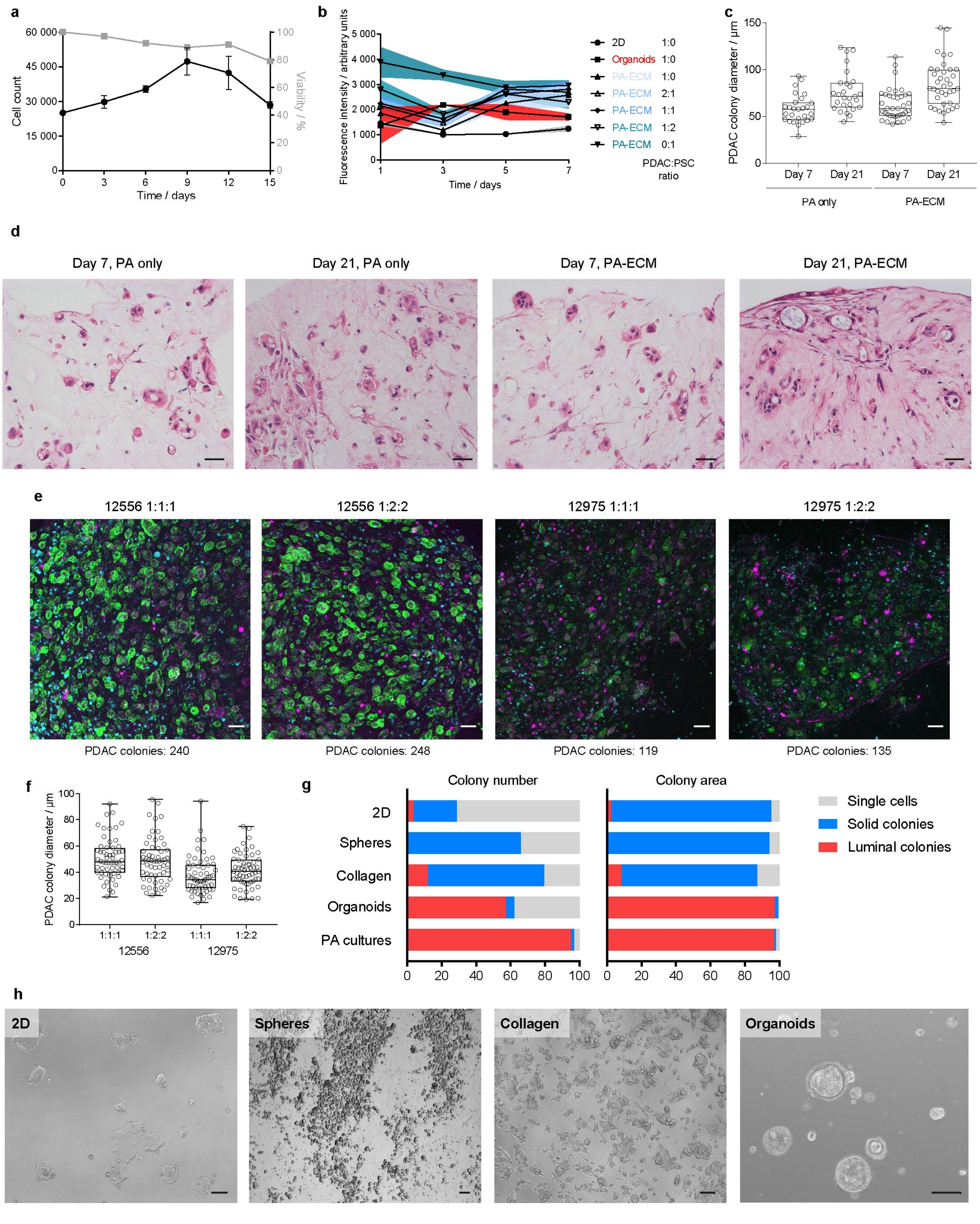

Supplementary Figure 1. *Ex vivo* PDAC colony characterization. a) Cell counts and viability of PA-ECM cultures over a two-week period (mean ± SD; n = 3 biological replicates). **b)** Metabolic activity of 2D, PA-ECM and organoid cultures as a measure of resazurin reduction (mean ± SD; n = 8 biological replicates). **c)** Diameter of PDAC colonies cultured in PA hydrogels with and without ECM proteins for 7 and 21 days. Box plots indicate range, interquartile range and median. n = 27 (PA) and 37 (PA-ECM) randomly sampled colonies across three gels of each type. **d)** H&E stains of PDAC colonies grown in PA hydrogels assembled with and without ECM proteins for 7 and 21 days. Scale bar: 100 µm. **e)** 7-day triple cultures in PA-ECM from two different patients (12556 and 12707) with different proportions of PDAC:PSC:macrophages. Cells were stained for EpCAM (green), α-SMA (magenta) and CD68 (cyan). The number of colonies in each field of view is indicated under the images. Scale bar: 100 µm. **f)** Size distribution of the PDAC colonies shown on panel d. Box plots indicate range, interquartile range and median. **g)** Number and relative area of PDAC colonies cultured in various *ex vivo* conditions for 7 days. **h)** Brightfield micrograph of PDAC cells cultured in various *ex vivo* conditions for 7 days. Scale bar: 100 µm.

a **b**

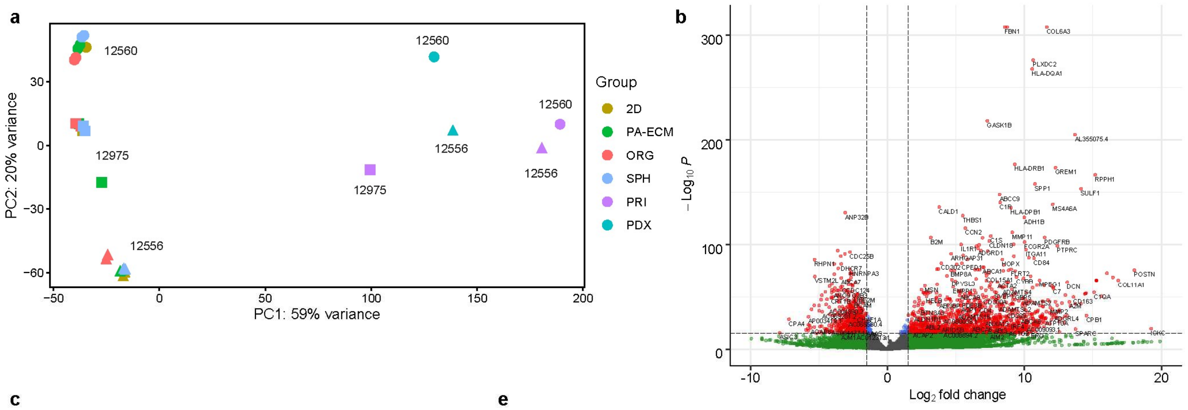

c

Basal	Classical	Odds	Basal / classical 12556	12560	12975
CD109	GPR160	2.38	5.28	3.02	6.58
SLC2A1	AGR2	3.39	1.84	1.07	0.15
KRT16	SLC44A4	1.68	0.21	0.02	0.81
CTSV	TMEM45B	4.17	0.08	0.13	0.35
KRT6A	BCAS1	2.01	0.29	0.04	1.81
B3GNT5	VSIG2	1.51	2.97	1.98	1.65
MET	TFF3	2.06	5.54	18.35	121.33
CHST6	PLA2G10	2.24	1.32	0.87	1.41
SERPINB5	HPGD	2.13	3.11	1.40	1.09
DCBLD2	PLS1	4.07	1.45	0.92	1.13
IL20RB	FAM3D	3.79	0.08	0.47	9.00
PPP1R14C	SYTL2	4.85	0.16	0.05	0.04
NAB1	PLEKHA6	1.50	1.58	3.37	2.12
MSLN	CAPN9	4.83	6.97	20.78	89.00

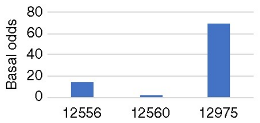

e

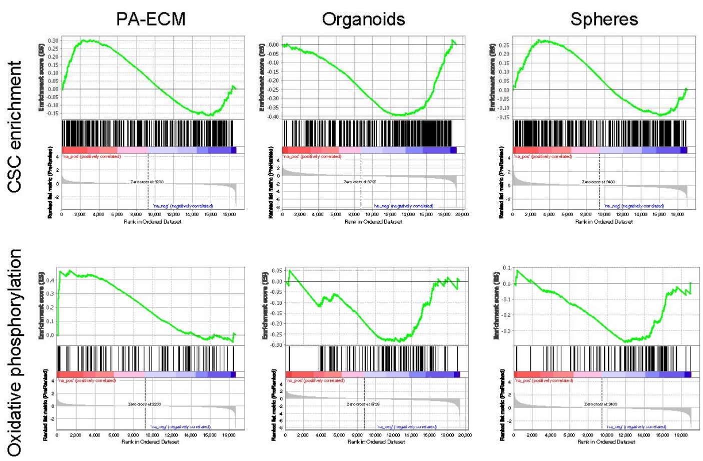

d

Basal	Classical	Odds	12556 PA-ECM	12556 ORG	12556 SPH	12560 PA-ECM	12560 ORG	12560 SPH	12975 PA-ECM	12975 ORG	12975 SPH
CD109	GPR160	2.4	45.60	16.33	62.94	5.89	4.68	0.57	23.05	15.48	18.12
SLC2A1	AGR2	3.4	15.24	16.80	5.31	1.96	4.53	0.63	58.42	>100	13.54
KRT16	SLC44A4	1.7	7.66	1.70	6.66	0.15	0.16	0.04	0.77	0.29	0.54
CTSV	TMEM45B	4.2	1.92	2.32	2.94	0.11	0.13	0.09	0.78	0.17	0.41
KRT6A	BCAS1	2	9.93	7.04	2.90	0.14	14.34	0.02	5.34	27.04	0.70
B3GNT5	VSIG2	1.5	4.59	14.00	1.91	1.13	2.20	0.39	23.22	47.96	19.46
MET	TFF3	2.1	>100	>100	>100	56.87	15.46	2.71	>100	>100	>100
CHST6	PLA2G10	2.2	27.22	16.78	27.24	7.21	77.18	4.17	>100	>100	>100
SERPINB5	HPGD	2.1	14.16	5.45	4.75	0.52	0.45	0.07	27.31	19.03	23.62
DCBLD2	PLS1	4.1	8.70	9.09	9.40	4.01	9.36	0.67	22.09	23.47	15.13
IL20RB	FAM3D	3.8	25.09	69.25	6.46	5.30	11.51	0.07	3.60	6.42	0.24
PPP1R14C	SYTL2	4.9	0.30	0.57	0.17	0.49	1.71	0.15	0.21	0.18	0.09
NAB1	PLEKHA6	1.5	1.11	1.22	0.62	0.73	1.22	0.40	0.74	0.88	0.59
MSLN	CAPN9	4.8	>100	>100	>100	30.99	>100	11.74	>100	>100	>100
		R	*0.712*	*0.681*	*0.710*	*0.688*	*0.583*	*0.564*	*0.316*	*0.283*	*0.336*

f

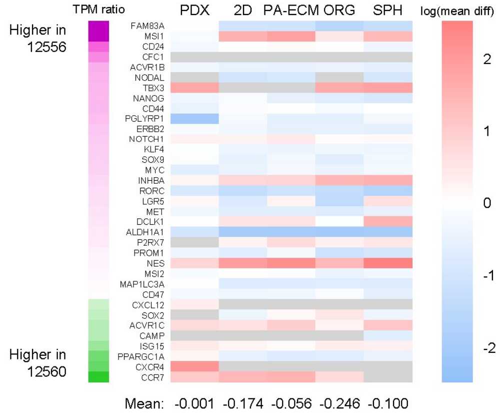

Supplementary Figure 2. Transcriptional profiles of *ex vivo* models of PDAC. a) Principal component analysis of the transcriptome of primary tumours, PDX and *ex vivo* models from patients 12556, 12560 and 12975. **b)** Volcano plot showing gene enrichment in primary tumours compared to organoid monocultures, with correction for inter-patient differences. **c)** Transcriptomic subtyping of primary tumours from patients 12556, 12560 and 12975 using the classifier odds calculated by Moffitt *et al.*[1] **d)** Classifier odds for patient-matched *ex vivo* models of PDAC and their correlation coefficients (*R*) vs primary. **e)** Gene set enrichment analysis of 3D *ex vivo* models with respect to 2D monolayers. Shown are the plots for Lytle's CSC enrichment gene list[2] and the KEGG oxidative phosphorylation gene list. **f)** Heatmap of the log mean difference between each model and the primary tumours for the ratio of CSC marker expression between patients 12556 and 12560. Genes for which no transcripts were detected in at least one sample are shown in grey.

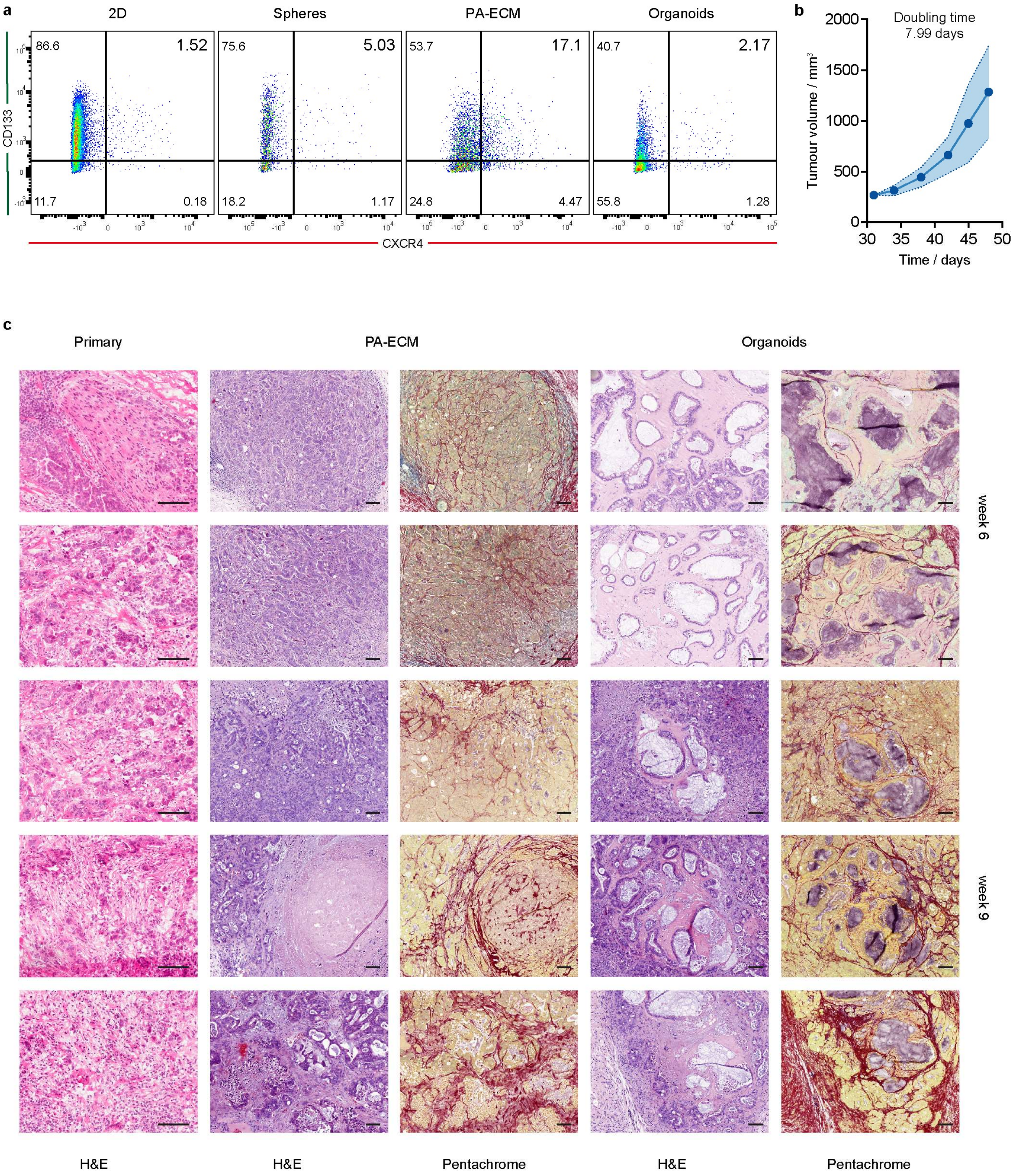

Supplementary Figure 3. Stemness and tumour formation from PA-ECM cultures. a) Representative flow cytometry plots of CD133+/CXCR4+ PDAC cells (12556) from 2D, sphere, PA-ECM and organoid cultures. **b)** Log phase growth curve for the PDX tumour corresponding to patient 12560. Shaded area denotes standard deviation around mean. **c)** H&E and pentachrome staining of PA-ECM culture and organoid-derived tumours grown in nude mice for 6 or 9 weeks. Scale bar: 100 µm.

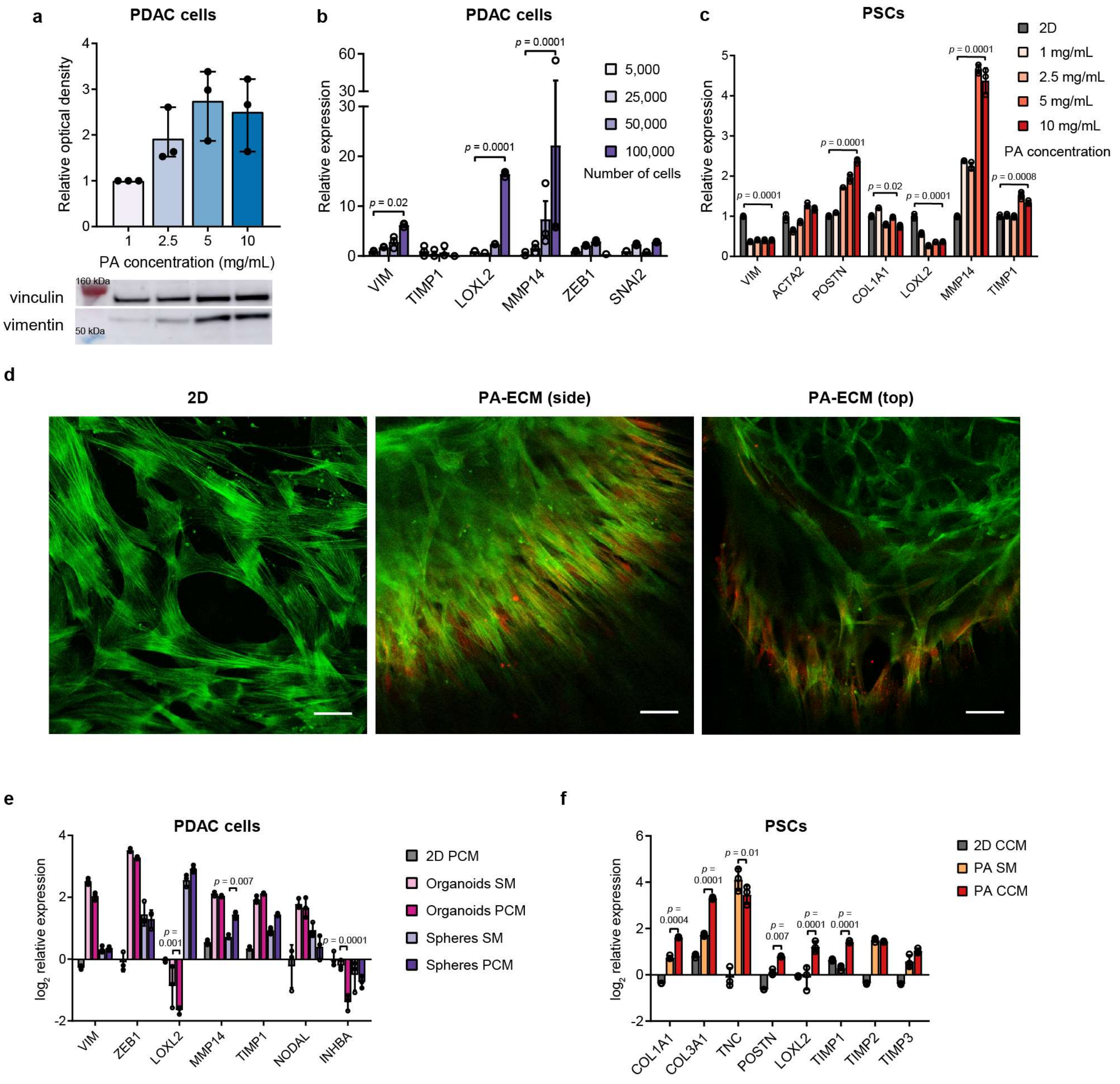

Supplementary Figure 4. PDAC and PSC plasticity in PA-ECM. a) Western blot analysis of vimentin expression in PDAC cells (12560) as a function of PA concentration (mean ± range; n = 3 biological replicates). **b)** Gene expression of PDAC cells (12560) grown in PA-ECM with increasing cell density (mean ± SD; n = 3 biological replicates). **c)** Gene expression of PSCs as a function of PA concentration relative to 2D controls (mean ± SD; n = 3 biological replicates). **d)** Immunofluorescence images of primary PSCs (green, phalloidin) expressing MMP14 (red) cultured for 7 days in 2D and PA-ECM. Scale bars: 50 µm. **e)** Log_2 normalized gene expression of PDAC cells (12560) cultured as organoids and spheres in sphere medium (SM) or PSC-conditioned medium (PCM) relative to the 2D SM baseline (mean ± SD; n = 3 biological replicates). **f)** Log_2 normalized gene expression of PSCs cultured in PA hydrogels in sphere medium (SM) or cancer cell-conditioned medium (CCM) relative to the 2D SM baseline (mean ± SD; n = 3 biological replicates).

Multiple comparisons in were performed by two-way ANOVA with Bonferroni correction.

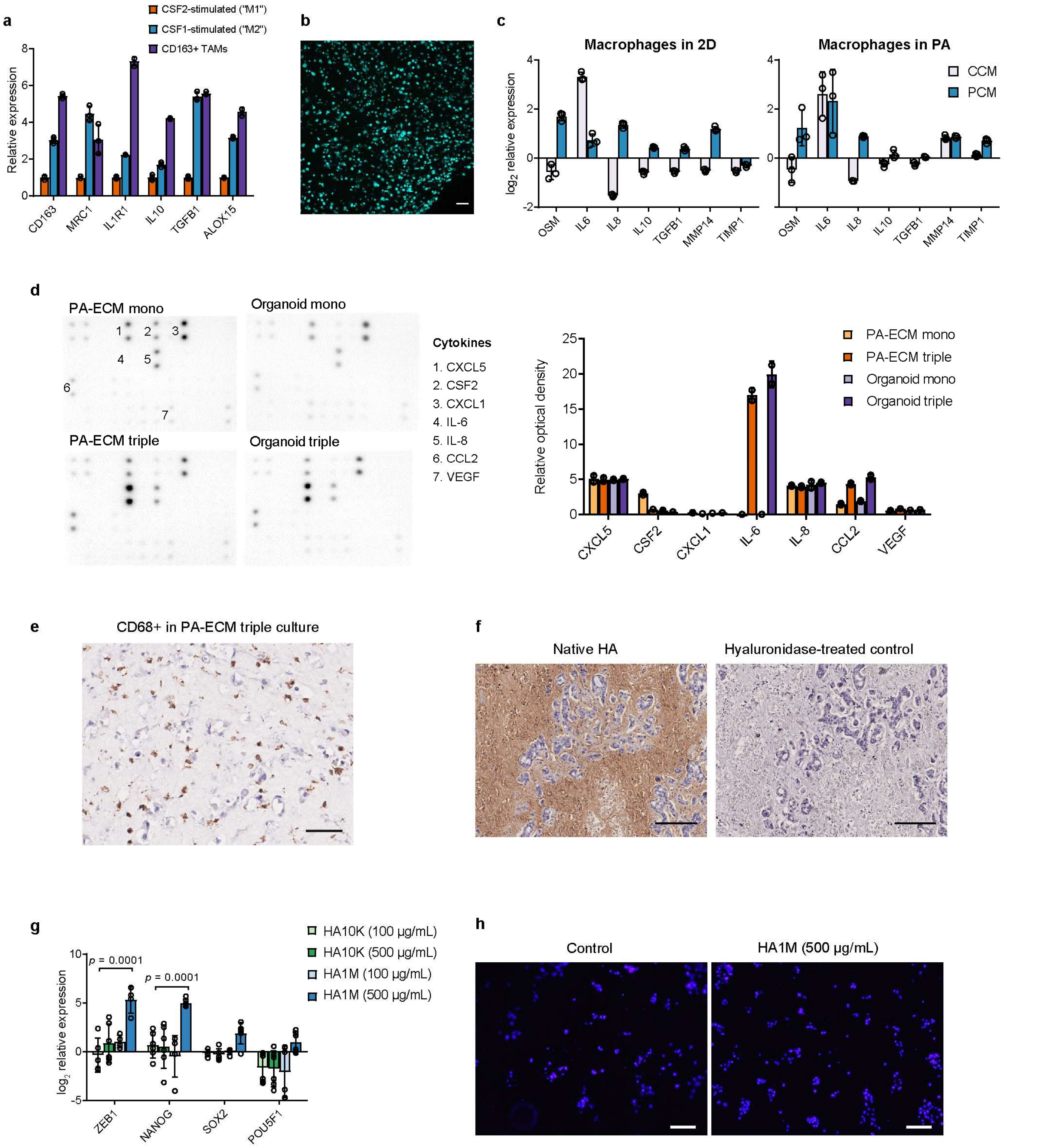

Supplementary Figure 5. Cytokines and hyaluronan in PA-ECM. a) Normalized gene expression of CSF2- and CSF1-stimulated macrophages derived from circulating monocytes as compared to CD163+ tumour-associated macrophages (TAMs) flow sorted from PDAC tumours (mean ± SD; n = 3 biological replicates). **b)** Projection of CD68+ macrophages in 7-day PA monocultures. Scale bar: 100 µm. **c)** Log_2 normalized gene expression of macrophages cultured in 2D and PA hydrogels in cancer cell-conditioned medium (CCM) or PSC-conditioned medium (PCM) relative to the sphere medium (SM) baseline (mean ± SD; n = 3 biological replicates). **d)** Cytokines detected in conditioned media from PDAC (12560) mono- and triple cultures grown in PA-ECM and as organoids. The bar chart represents the densitometric readings of the arrays shown on the left (mean ± SD; n = 2 replicate antibody assays per sample). **e)** Immunohistochemical stain of CD68+ macrophages in 7-day PA triple cultures. Scale bar: 100 µm. **f)** Representative staining of hyaluronan in PDX tissue (12560) compared to the hyaluronidase-treated control. Scale bars: 200 µm. **g)** Log_2 normalized gene expression of PDAC cells (12560) cultured in PA hydrogels as a function of the concentration of low (10 kDa) and high (1 MDa) molecular mass hyaluronan (mean ± SD; n = 5 biological replicates). Multiple comparisons were performed by two-way ANOVA with Bonferroni correction. **h)** Representative immunofluorescence images of invaded PDAC cells (12560) in the presence of high (1 MDa) molecular mass hyaluronan compared to the control without hyaluronan. Scale bars: 500 µm.

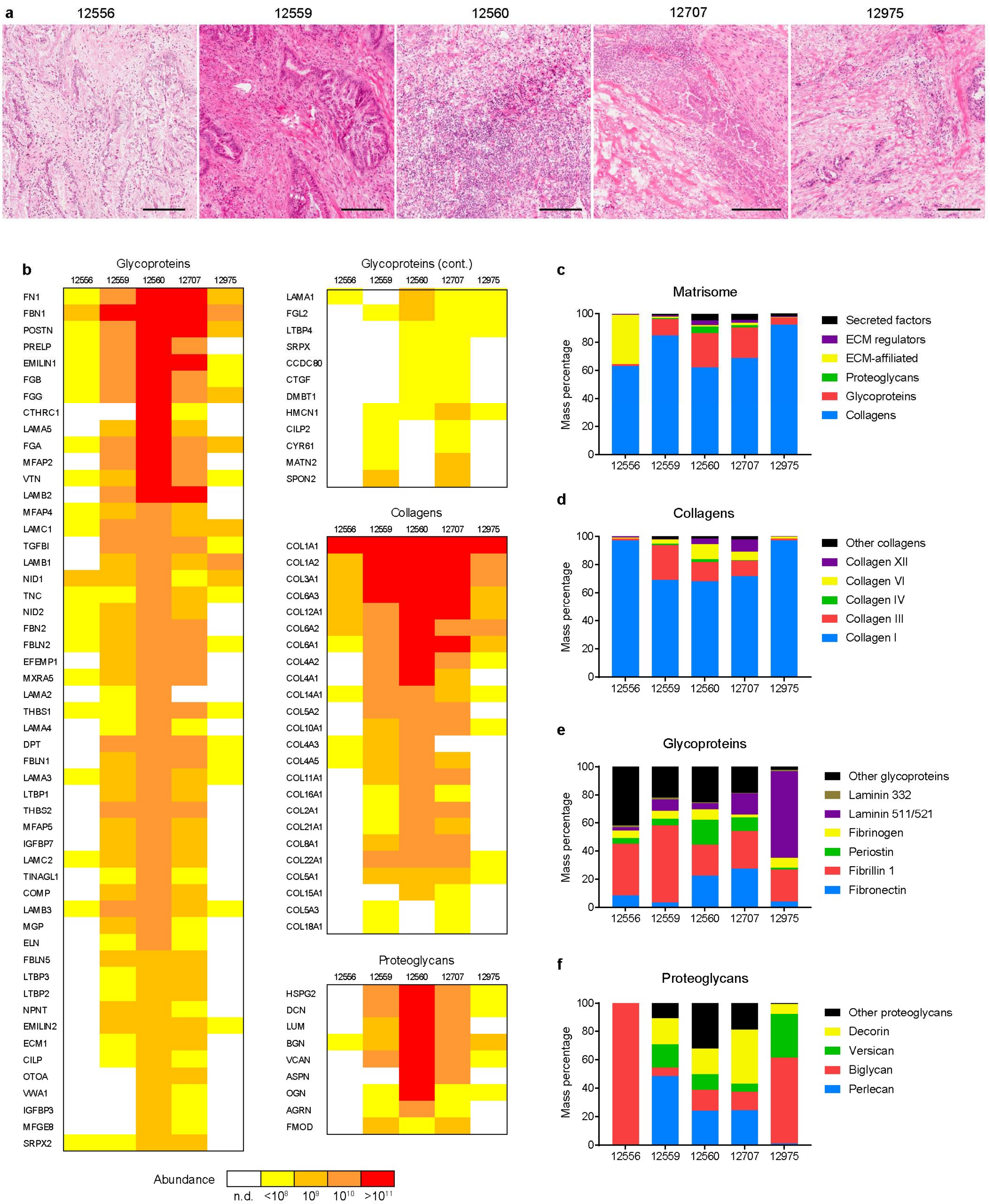

Supplementary Figure 6. Inter-patient PDAC matrisome heterogeneity. a) Representative H&E micrographs of primary tumour tissues of origin. Scale bars: 100 µm. **b)** Abundance heatmap of matrisome proteins detected in two or more patients, with two or more unique peptides each, shown in order of abundance by category. **c)** Mass distribution of the main matrisome categories across patients. **d)** Mass distribution of collagens across patients. **e)** Mass distribution of glycoproteins across patients. **f)** Mass distribution of proteoglycans across patients.

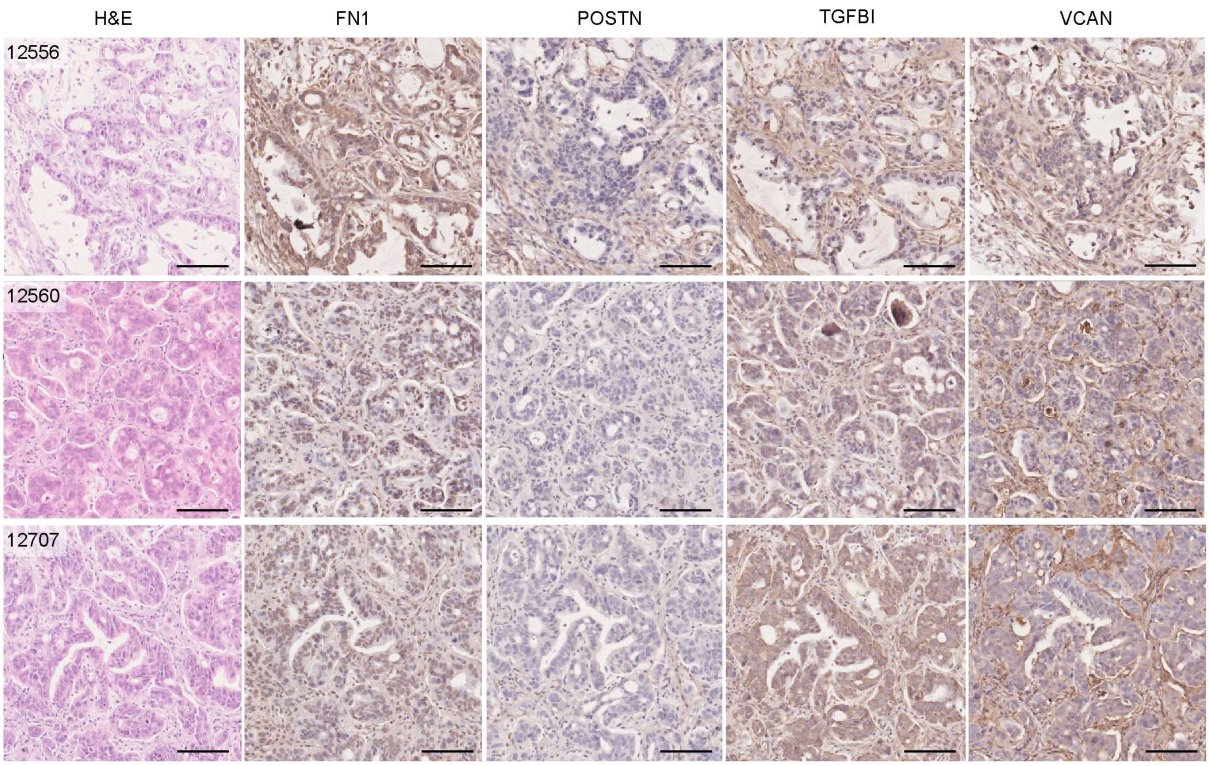

Supplementary Figure 7. Immunohistochemical validation of core ECM proteins in PDAC tissues. Representative H&E stains and IHC stains of fibronectin (FN1), periostin (POSTN), transforming growth factor beta-induced (TGFβI) and versican (VCAN) in consecutive slices of PDX tissues. Scale bars: 100 μm.

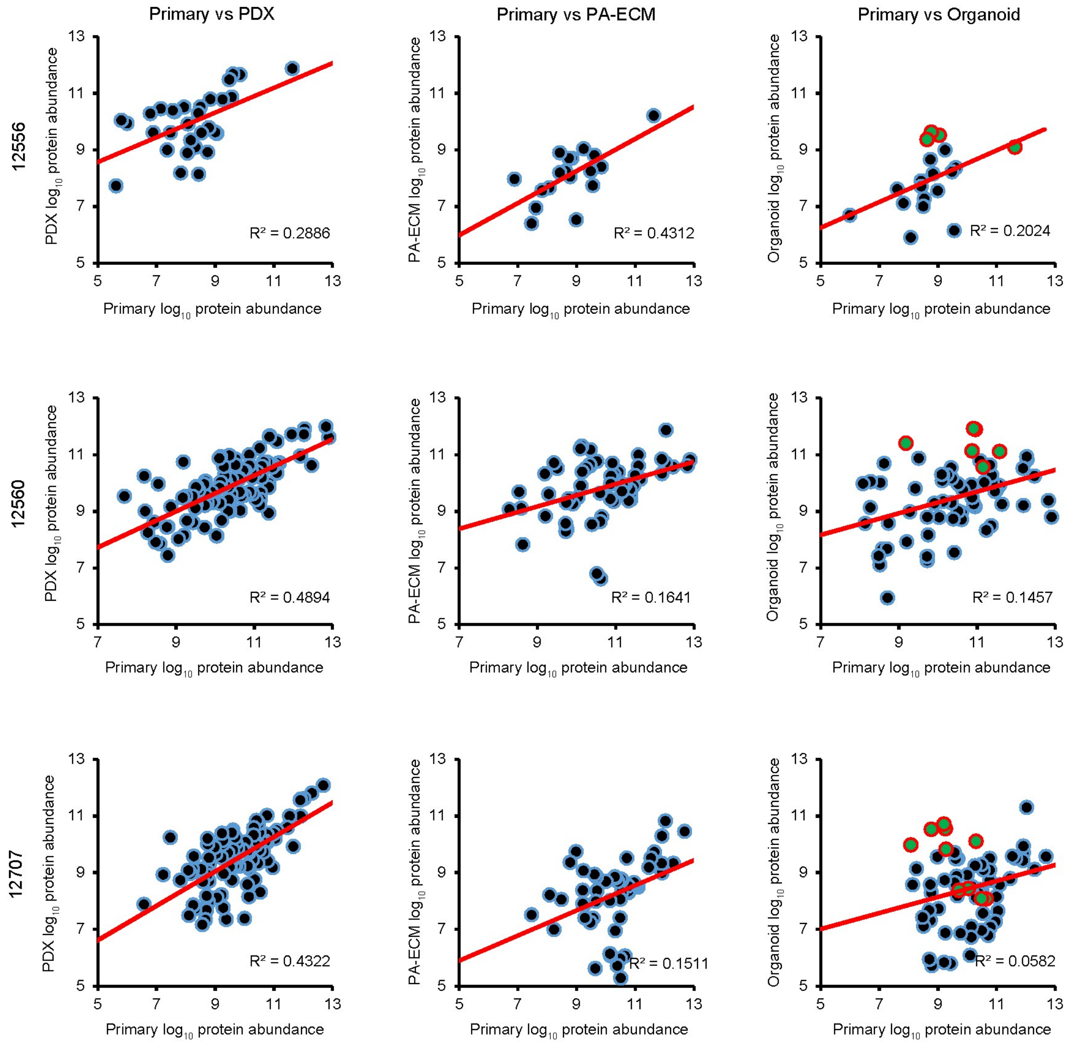

Supplementary Figure 8. Core matrisome abundance correlations. The scatter plots represent the correlation between the core matrisome protein abundances in primary tumours and their corresponding PDX, PA-ECM and organoid models from patients 12556, 12560 and 12707. Overrepresented Matrigel components are shown in red/green circles.

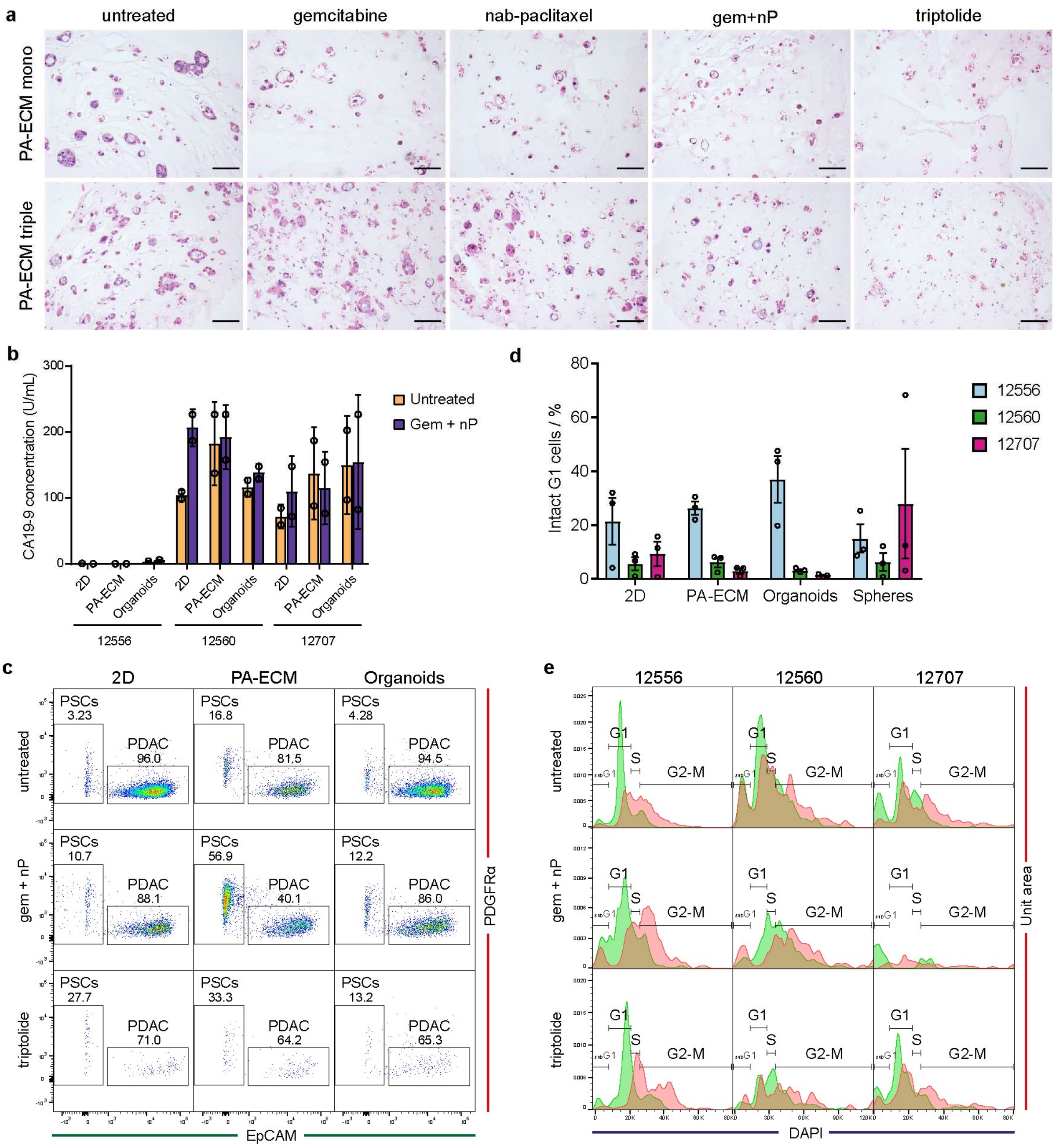

Supplementary Figure 9. Measurement of drug response in PA-ECM cultures. a) H&E staining of PA-ECM mono- and triple cultures treated with gemcitabine, nab-paclitaxel and triptolide. Scale bars: 100 µm. **b)** CA19-9 concentration in conditioned media from PDAC cells (12556, 12560, 12707) treated with gemcitabine/nab-paclitaxel (mean ± SD; n = 2 biological replicates). **c)** Representative flow cytometry plots of co-cultures of PDAC cells (12707) and PSCs in 2D, PA-ECM and organoids, untreated and treated with gemcitabine/nab-paclitaxel and triptolide. **d)** Response to triptolide by PDAC cells (12556, 12560, 12707) cultured in 2D, PA-ECM, organoids and spheres as measured by flow cytometry. Percentages represent the proportion of cells that were both intact and in G1 phase compared to untreated controls (mean ± SD; n = 3 biological replicates). **e)** Cell cycle profiles of CD133+/CXCR4+ (red) and CD133-/CXCR4- (green) cells (12556, 12560, 12707) cultured in PA-ECM and treated with gemcitabine/nab-paclitaxel and triptolide.

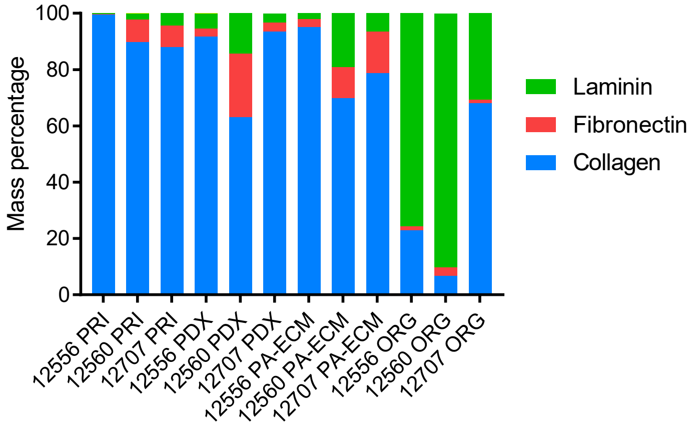

Supplementary Figure 10. Ratio of collagen to fibronectin to laminin across samples. Proportions represent the relative abundances measured by mass spectrometry.

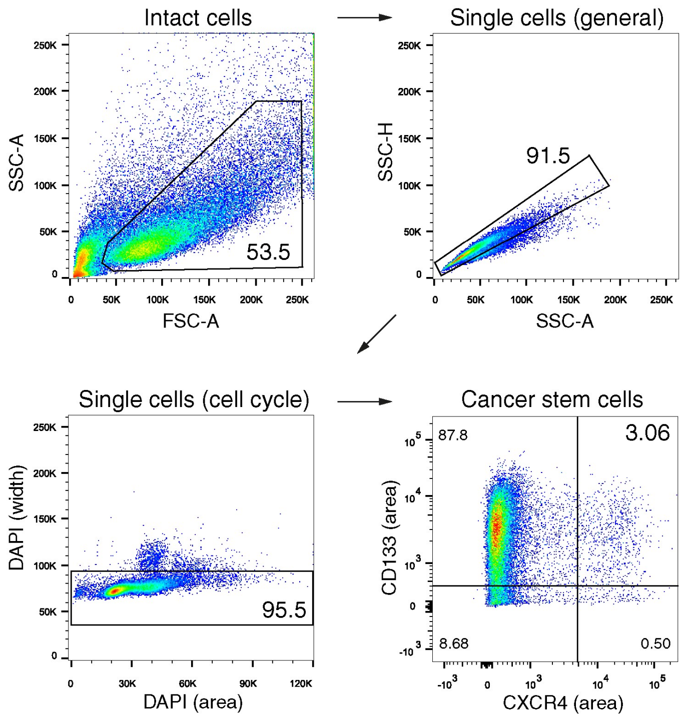

Supplementary Figure 11. Flow cytometry gating strategy. SSC = side scatter, FSC = forward scatter.

Supplementary Table 1. Advantages and disadvantages of PA-ECM hydrogels compared to various established PDAC models.

Model	Advantages of PA-ECM hydrogels	Disadvantages of PA-ECM hydrogels	Ref
Genetically engineered mouse models (e.g. KPC)	Fewer resources and less time required in PA-ECM, which also enables the study human cells with a wider range of genetic backgrounds.	Cannot study tumour formation (e.g. longitudinally) and lack of *in vivo* tissue organization.	3
Patient-derived xenografts	Fewer resources and less time required in PA-ECM, which supports the seeding of PDX-derived cells.	Lack of *in vivo* tissue and cell type heterogeneity.	4
Tissue explants	PA-ECM gels are more versatile in terms of cells and composition, and suitable for longer experiments in less restricted conditions.	Tissue architecture and organization, including stromal cell types, not as well reflected as in explants.	5
Decellularized tissues	Self-assembly is more tuneable and versatile, as well as amenable to a wider range of downstream analyses; reduced variability between batches.	Tissue architecture and organization, especially most matrix components, not as well reflected as in decellularized tissues.	6
Matrigel	PA-ECM gels are tuneable in terms of composition and physical properties, overall stiffer and combinable with a wider range of ECM molecules.	Not as standardized for the directed differentiation of organoids, especially for tissues that are laminin-rich, like the brain, or those with defined niches, like the intestinal crypt.	7
Collagen gels	More versatile composition of PA-ECM and mechanically tuneable without the need for crosslinking; user-defined co-assembly with collagen and other ECM proteins is possible.	More expensive and labour-intensive, although they can be combined with collagen, PAs are not part of actual tissues.	8
Alginate	PA-ECM constructs are more representative of the tissue matrix and tuneable than alginate hydrogels (which are not fibrillar).	Hydrogel preparation and cell recovery is easier in alginate, which is also a cheaper material.	9
PDMS	PA-ECM gels support cell attachment and can be assembled with numerous proteins found in tissues.	PDMS can be micropatterned and enables the formation of microfluidic devices; PA-ECM may be compatible with such approaches.	10
PEG	PA-ECM gels are fibrillar, support protein co-assembly and enhance cell attachment compared to PEG.	PEG gels support a wide stiffness range and a more validated use in the literature and in medical settings.	11
Polyacrylamide	PA-ECM gels are non-toxic (unlike acrylamide) and support 3D cultures.	Polyacrylamide facilitates the modification of 2D mechanical properties with ease.	12
Methacrylated hyaluronan	Tuneability of PA-ECM does not require radical polymerization and supports assembly with a wider range of ECM molecules.	MeHA hydrogels support temporal gradients via cross-linking and are more biodegradable and amenable to direct conjugation with various epitopes.	13
Methacrylated gelatin	Tuneability of PA-ECM does not require radical polymerization and supports assembly with a wider range of ECM molecules.	GelMa has more tuneable mechanics, is MMP-responsive and has wider use in microfabrication.	14
Other self-assembling peptides	Co-assembly with ECM enables recapitulation of the matrix composition and architecture and control over niche-related signalling.	Self-assembling peptide hydrogels are cheaper and commercially available and may support enhanced attachment via presentation of epitopes such as RGD.	15

Supplementary Table 2. Details of patient-derived PDAC tissues.

Patient	Gender	Age	pT	pN	M	Stage	Histology	Mutations
12556	Male	67	3	1	0	IIB	PDAC	*KRAS, TP53*
12559	Female	80	3	1	0	IIB	PDAC	*KRAS, TP53, SMAD4, CDKN2A*
12560	Male	60	3	1	0	IIB	PDAC	*KRAS*
12707	Male	60	3	1	0	IIB	PDAC	*KRAS*
12975	Female	75	3	0	0	IIA	IPMN/PDAC	*KRAS*

Macrophages were obtained from a mixed pool of male and female healthy donors aged 26 to 50 years old.

Supplementary Table 3. Details of antibodies used for immunofluorescence (IF), Western blotting (WB), flow cytometry (FC) and immunohistochemistry (IHC).

Antigen	Clone	Conjugate	Brand	Product number	Application	Dilution
EpCAM (CD326)	VU1D9	Alexa 488	Cell Signaling	5198S	IF	1:1000
Ki-67	B126.1	-	Abcam	ab8191	IF	1:1000
EpCAM (CD326)	-	-	Abcam	ab71916	IF	1:1000
α-SMA	1A4	FITC	Sigma-Aldrich	F3777	IF	1:1000
Vimentin	EPR3776	-	Abcam	ab92547	IF, WB	1:1000
Vinculin	V284	-	Sigma-Aldrich	SAB4200080	WB	1:2000
EpCAM (CD326)	9C4	PE	BioLegend	324206	FC	1:20
PDGFRα (CD140a)	αR1	Alexa 647	BD Biosciences	562798	FC	1:20
Prominin (CD133)	AC133	PE	Miltenyi Biotec	130-080-801	FC	1:40
CXCR4 (CD184)	12G5	APC	BioLegend	306510	FC	1:20
CD68	PG-M1	-	Dako	M0876	IHC	1:50
Hyaluronan	HABP2	Biotin	Merck	385911	IHC	1:100
Fibronectin	-	-	Sigma-Aldrich	F3648	IHC	1:600
Periostin	-	-	Sigma-Aldrich	HPA012306	IHC	1:200
TGF-βI	-	-	Sigma-Aldrich	HPA017019	IHC	1:700
Versican	-	-	Sigma-Aldrich	HPA004726	IHC	1:250
Ki-67	MIB-1	-	Dako	M7240	IHC	1:1000
Cleaved caspase 3	5AIE	-	Cell Signaling	9664S	IHC	1:50

Supplementary Table 4. Gene-specific primer pairs for RT-qPCR.

Gene	Forward primer	Reverse primer	Amplicon bp
ACTA2	ATCCCCGGGACTAAGACGGGAATC	AAGCCGGCCTTACAGAGCCCA	111
ALDH1A1	GCTTCCGAGAGGGGGCGACT	TCCATTGTCGCCAGCAGCAGA	77
ALOX15	CTTCAAGCTTATAATTCCCCAC	GATTCCTTCCACATACCGATAG	280
CD44s	TCCAACACCTCCCAGTATGACA	GGCAGGTCTGTGACTGATGTACA	83
CD44v6	GGAACAGTGGTTTGGCAACAG	TTGGGTGTTTGGCGATATCC	51
CD163	AGCATGGAAGCGGTCTCTGTGATT	AGCTGACTCATTCCCACGACAAGA	138
COL1A1	TCTGCGTCTGCGACAACGGC	GGTGACTCTGAGCCGTCGGG	127
COL3A1	CGCCCTCCTAATGGTCAAGG	TTCTGAGGACCAGTAGGGCA	161
IL6	TCGAGCCCACCGGGAACGAA	GGACCGAAGGCGCTTGTGGA	86
IL8	TGACTTCCAAGCTGGCCGTGG	ACTGCACCTTCACACAGAGCTGC	70
IL10	ACCTGCCTAACATGCTTCGAG	AACCCTTAAAGTCCTCCAGCAA	123
IL1R1	CCTGCTATGATTTTCTCCCAATAAA	CACAAAAATATCACAGTCAGAGGTAGAC	113
INHBA	AAAGCTTCATGTGGGCAAAG	AATCTCGAAGTGCAGCGTCT	157
ITGB1	AGAAGGGTTGCCCTCCAGAT	TGCTGTTCCTTTGCTACGGT	95
KLF4	TCTCCACGTTCGCGTCTGGC	TCCCGCCAGCGGTTATTCGG	79
LOXL2	GGCACCGTGTGCGATGACGA	GCTGCAAGGGTCGCCTCGTT	170
MMP14	ATGGGCCCAACATCTGTGA	CCAGCGCTCCTTGAAGACA	80
MRC1	ACAACAAAAGCTGACACAAGGA	AGGACAGACCAGTACAATTCAG	379
MYC	CCCGCTTCTCTGAAAGGCTCTC	CTCTGCTGCTGCTGCTGGTAG	198
NANOG	AGAACTCTCCAACATCCTGAACCT	TGCCACCTCTTAGATTTCATTCTCT	84
NODAL	AGCATGGTTTTGGAGGTGAC	CCTGCGAGAGGTTGGAGTAG	160
OSM	CGCTGCTCAGTCTGGTCCTT	CGCGGTACTCTTTCGAGCA	84
POSTN	TAGAGCAGCTGCCATCACATC	TCTAGGACACCTCGTGGAAGT	114
POU5F1	CTTGCTGCAGAAGTGGGTGGAGGAA	CTGCAGTGTGGGTTTCGGGCA	169
RPS13	TCGGCTTTACCCTATCGACGCAG	ACGTACTTGTGCAACACCATGTGA	153
SNAI2	ATGCCGCGCTCCTTCCT	TGTGTCCAGTTCGCT	72
SOX2	AGAACCCCAAGATGCACAAC	CGGGGCCGGTATTTATAATC	154
TGFB1	GCGTGCTAATGGTGGAAACC	GGAGAGCAACACGGGTTCAG	125
TIMP1	GGGGATGCCGCTGACATCCG	CTCGCTGCGGTTGTGGGACC	87
TIMP2	AAGAACATCAACGGGCACCA	CGAGAAACTCCTGCTTGGGG	106
TIMP3	GTACCGAGGCTTCACCAAGA	CCCACCTCTCCACGAAGTTG	167
TNC	TAGTGGTCAAGTGGGAGGGG	AGCTTTTCCCAAGTGTGTTCA	94
VIM	GACAATGCGTCTCTGGCACGTCTT	TCCTCCGCCTCCTGCAGGTTCTT	236
ZEB1	GATGATGAATGCGAGTCAGATGC	CTGGTCCTCTTCAGGTGCC	117

SUPPLEMENTARY METHODS

Peptide synthesis and purification

The C_{16}-$V_3A_3E_3$ (E3 PA) peptide amphiphile was synthesized by solid-phase peptide synthesis on a Liberty Blue instrument (CEM, UK) with a Rink amide resin. Fmoc-protected amino acids were coupled by 1-hydroxybenzotriazole and *N,N'*-diisopropyl-carbodiimide in *N,N*-dimethylformamide, followed by addition of the palmitoyl tail and final cleavage with 95% trifluoracetic acid. PAs were purified by reverse-phase HPLC as previously reported [16].

Electron microscopy

For transmission electron microscopy, 20 µL PA solution (0.1 mg/mL in HEPES) was placed on a copper grid, stained with 2% uranyl acetate, air dried and imaged on a JEOL 2010 microscope as previously described [17]. For scanning electron microscopy, 10 mg/mL E3 PA hydrogels were prepared in $CaCl_2$, fixed with 2.5% glutaraldehyde, lyophilized and sputter coated with gold prior to imaging on a FEI Inspect F50 microscope [17].

Circular dichroism spectroscopy

Circular dichroism (CD) of PA in solution (0.1 mg/mL in HEPES) was measured on a Chirascan spectrometer (Applied Photophysics, UK). Samples were run in triplicate within a scan range from 190 to 260 nm. Spectroscopic data were analysed on Pro-Data Viewer (Applied Photophysics, UK). Blanks (HEPES buffer only) were run to subtract noise from readings. Further noise reduction was achieved by applying a smoothing factor of 10.

Tissue and hydrogel stiffness measurements

The stiffness of PDX tissue from PDAC patient 12707 was measured by atomic force microscopy on a Nanowizard 4 (JPK Instruments, Germany) mounted on an IX81 inverted microscope (Olympus, Japan). Analysis was performed in quantitative imaging mode using an MLCT cantilever (Bruker, US) with a spring constant of 0.07 N/m and a pyramidal tip with a diameter of 20 nm and a front angle of 15 ± 2.5°. Force curves were acquired from a 5 µm tissue section with a setpoint force of 2 nN across 4000 nm and a pixel time of 100 ms. Nanowizard 4 software (JPK Instruments, Germany) was used to calculate the Young's modulus by fitting the contact region of the approach curve with the Hertz contact model.

The stiffness of PA-ECM (50 µL, 10 mg/mL) and Matrigel (50 µL, 9 mg/mL) was measured on a Discovery HR-3 rheometer (TA Instruments, USA). Frequency sweeps were run from 0.1 to 100 Hz.

ECM enrichment and mass spectrometry preparation

For ECM enrichment, primary tumour and PDX samples were microtome-sectioned in advance to enable solubilization, while hydrogels were mechanically dissociated by pipetting in buffer C. Cellular compartments were sequentially separated with buffers C, N, M and CS from the CNMCS compartmental protein extraction kit (BioChain, US) as previously described [18]. The only modification to this protocol was the use of 20% buffer CS and 80% buffer M instead of 100% buffer CS to prevent depletion of ECM proteins. This modification has been previously used for PDAC ECM enrichment [19]. The resulting ECM-enriched pellets were solubilized in 8 M urea in 20 mM HEPES buffer and normalized for protein content (80 µg) before undergoing sequential reduction, alkylation and digestion with Lys-C protease and trypsin (Life Technologies, UK) as previously described [18]. Ultra-performance liquid chromatography was performed on a NanoAcquity system (Waters, USA) to separate peptides before analysis on the LTQ-Orbitrap XL mass spectrometer (Thermo Scientific, USA).

Enzyme-linked immunosorbent assay

Secretion of cytokines and CA19-9 in conditioned medium from 3D cell cultures after 10 days was assayed using a cytokine array (Abcam, UK) and a pre-coated kit (Life Technologies, UK), respectively.

References

1. Moffitt, R. A. *et al.* Virtual microdissection identifies distinct tumor- and stroma-specific subtypes of pancreatic ductal adenocarcinoma. *Nat. Genet.* **47**, 1168–1178 (2015).
2. Lytle, N. K. *et al.* A multiscale map of the stem cell state in pancreatic adenocarcinoma. *Cell* **177**, 572-586.e22 (2019).
3. Hingorani, S. R. *et al.* Trp53R172H and KrasG12D cooperate to promote chromosomal instability and widely metastatic pancreatic ductal adenocarcinoma in mice. *Cancer Cell* **7**, 469–483 (2005).
4. Hidalgo, M. *et al.* Patient-derived xenograft models: An emerging platform for translational cancer research. *Cancer Discov.* **4**, 998–1013 (2014).
5. Majumder, B. *et al.* Predicting clinical response to anticancer drugs using an ex vivo platform that captures tumour heterogeneity. *Nat. Commun.* **6**, 1–14 (2015).
6. Gaetani, R. *et al.* Evaluation of different decellularization protocols on the generation of pancreas-derived hydrogels. *Tissue Eng. Part C Methods* **24**, ten.TEC.2018.0180 (2018).
7. Boj, S. F. *et al.* Organoid models of human and mouse ductal pancreatic cancer. *Cell* **160**, 324–338 (2015).
8. Brown, R. A. In the beginning there were soft collagen-cell gels: Towards better 3D connective tissue models? *Exp. Cell Res.* **319**, 2460–2469 (2013).
9. Chaudhuri, O. *et al.* Extracellular matrix stiffness and composition jointly regulate the induction of malignant phenotypes in mammary epithelium. *Nat. Mater.* **13**, 970–978 (2014).
10. Zhang, W., Choi, D. S., Nguyen, Y. H., Chang, J. & Qin, L. Studying cancer stem cell dynamics on PDMS surfaces for microfluidics device design. *Sci. Rep.* **3**, 2332 (2013).
11. Loessner, D. *et al.* Bioengineered 3D platform to explore cell-ECM interactions and drug resistance of epithelial ovarian cancer cells. *Biomaterials* **31**, 8494–8506 (2010).
12. Zustiak, S., Nossal, R. & Sackett, D. L. Multiwell stiffness assay for the study of cell responsiveness to cytotoxic drugs. *Biotechnol. Bioeng.* **111**, 396–403 (2014).
13. Ondeck, M. G. & Engler, A. J. Mechanical characterization of a dynamic and tunable methacrylated hyaluronic acid hydrogel. *J. Biomech. Eng.* **138**, 021003 (2016).
14. Loessner, D. *et al.* Functionalization, preparation and use of cell-laden gelatin methacryloyl-based hydrogels as modular tissue culture platforms. *Nat. Protoc.* **11**, 727–746 (2016).
15. Betriu, N. & Semino, C. Development of a 3D co-culture system as a cancer model using a self-assembling peptide scaffold. *Gels* **4**, 65 (2018).
16. Berns, E. J. *et al.* A tenascin-C mimetic peptide amphiphile nanofiber gel promotes neurite outgrowth and cell migration of neurosphere-derived cells. *Acta Biomater.* **37**, 50–58 (2016).
17. O'Leary, L. E. R., Fallas, J. A., Bakota, E. L., Kang, M. K. & Hartgerink, J. D. Multi-hierarchical self-assembly of a collagen mimetic peptide from triple helix to nanofibre and hydrogel. *Nat. Chem.* **3**, 821–828 (2011).
18. Naba, A., Clauser, K. R. & Hynes, R. O. Enrichment of extracellular matrix proteins from tissues and digestion into peptides for mass spectrometry analysis. *J. Vis. Exp.* e53057 (2015). doi:10.3791/53057
19. Tian, C. *et al.* Proteomic analyses of ECM during pancreatic ductal adenocarcinoma progression reveal different contributions by tumor and stromal cells. *Proc. Natl. Acad. Sci. U. S. A.* **116**, 19609–19618 (2019).
