# Supplementary material for: Bioengineered 3D models of human pancreatic cancer recapitulate in vivo tumour biology
Source: Nat Commun. 2021 Sep 24;12:5623. doi: 10.1038/s41467-021-25921-9 (PMC8463670; doi:10.1038/s41467-021-25921-9)
Supplement: Supplementary file 2 — Description of Additional Supplementary Files [file 41467_2021_25921_MOESM2_ESM.docx]

**Bioengineered 3D models of human pancreatic cancer recapitulate *in vivo* tumour biology – Supplementary Data Legends**

David Osuna de la Peña, Sara Maria David Trabulo, Estelle Collin, Ying Liu, Shreya Sharma, Marianthi Tatari, Diana Behrens, Bruno Sainz, Mert Erkan, Rita T. Lawlor, Aldo Scarpa, Christopher Heeschen, Álvaro Mata, Daniela Loessner

**Supplementary Data 1.** Gene lists for differential expression analysis and summary of KEGG pathway enrichment.

**Supplementary Data 2.** Complete PDAC matrisome table of protein abundances.

**Supplementary Data 3.** Summary of statistical analysis. False positive risk (FPR) estimated using online FPR calculator (Colquhoun & Longstaff, 2017: http://fprcalc.ucl.ac.uk/) for a prior probability of 0.5.
