# Supplementary material for: Bioengineered 3D models of human pancreatic cancer recapitulate in vivo tumour biology
Source: Nat Commun. 2021 Sep 24;12:5623. doi: 10.1038/s41467-021-25921-9 (PMC8463670; doi:10.1038/s41467-021-25921-9)
Supplement: Supplementary file 6 — Reporting Summary [file 41467_2021_25921_MOESM6_ESM.pdf]

nature research

Corresponding author(s): Christopher Heeschen, Alvaro Mata, Daniela Loessner

Last updated by author(s): Jul 9, 2021

Reporting Summary

Nature Research wishes to improve the reproducibility of the work that we publish. This form provides structure for consistency and transparency in reporting. For further information on Nature Research policies, see our Editorial Policies and the Editorial Policy Checklist.

Statistics

For all statistical analyses, confirm that the following items are present in the figure legend, table legend, main text, or Methods section.

n/a	Confirmed	
☐	☒	The exact sample size (n) for each experimental group/condition, given as a discrete number and unit of measurement
☐	☒	A statement on whether measurements were taken from distinct samples or whether the same sample was measured repeatedly
☐	☒	The statistical test(s) used AND whether they are one- or two-sided<br>*Only common tests should be described solely by name; describe more complex techniques in the Methods section.*
☒	☐	A description of all covariates tested
☐	☒	A description of any assumptions or corrections, such as tests of normality and adjustment for multiple comparisons
☐	☒	A full description of the statistical parameters including central tendency (e.g. means) or other basic estimates (e.g. regression coefficient) AND variation (e.g. standard deviation) or associated estimates of uncertainty (e.g. confidence intervals)
☐	☒	For null hypothesis testing, the test statistic (e.g. F, t, r) with confidence intervals, effect sizes, degrees of freedom and P value noted<br>*Give P values as exact values whenever suitable.*
☒	☐	For Bayesian analysis, information on the choice of priors and Markov chain Monte Carlo settings
☒	☐	For hierarchical and complex designs, identification of the appropriate level for tests and full reporting of outcomes
☐	☒	Estimates of effect sizes (e.g. Cohen's d, Pearson's r), indicating how they were calculated

Our web collection on statistics for biologists contains articles on many of the points above.

Software and code

Policy information about availability of computer code

Data collection

Flow cytometry data: BD FACSDiva (Becton, Dickinson and Company, USA).
qPCR data: QuantStudio 7 (Applied Biosystems, UK).
Transcriptomic data: NovaSeq 6000 (Illumina, USA).
Proteomic data: LTQ-Orbitrap XL (Thermo Scientific, USA).
Atomic force microscopy: Nanowizard 4 (JPK Instruments, Germany).
IF images: Zeiss LSM 710 (Zeiss, Germany).
IHC images: Pannoramic 250 Flash III scanner (3DHISTECH, Hungary) and Ariol system (Leica Biosystems, Germany).

Data analysis

Flow cytometry: FlowJo v10.
Circular dichroism: Pro-Data Viewer (Applied Photophysics, UK).
Imaging data: ImageJ 1.52u (NIH, USA).
Proteomic data: Mascot (for protein identification), PESCAL (for quantification) and Microsoft Excel 2020 (for abundance analysis).
Transcriptomic data were mapped with kallisto v0.45.0 and processed with DESeq2 in R 3.5.3 (R Foundation, USA) and with GSEA 4.0.1.
All data were processed in Prism 7.05 (GraphPad, USA) and imported into Illustrator CC (Adobe, USA) for visualization.

For manuscripts utilizing custom algorithms or software that are central to the research but not yet described in published literature, software must be made available to editors and reviewers. We strongly encourage code deposition in a community repository (e.g. GitHub). See the Nature Research guidelines for submitting code & software for further information.

Data

Policy information about availability of data

All manuscripts must include a data availability statement. This statement should provide the following information, where applicable:

- Accession codes, unique identifiers, or web links for publicly available datasets
- A list of figures that have associated raw data
- A description of any restrictions on data availability

Raw and processed transcriptomic data are available at the GEO database under accession number GSE139184. Proteomic data are available at the PRIDE database under accession number PXD013254. All data that support the findings of this study are available within the Article, Supplementary information and Source data file.

Field-specific reporting

Please select the one below that is the best fit for your research. If you are not sure, read the appropriate sections before making your selection.

☒ Life sciences ☐ Behavioural & social sciences ☐ Ecological, evolutionary & environmental sciences

For a reference copy of the document with all sections, see nature.com/documents/nr-reporting-summary-flat.pdf

Life sciences study design

All studies must disclose on these points even when the disclosure is negative.

Sample size	Sample size was predetermined according to the type of experiment based on common practice. For most experiments, 10 gels per sample were prepared and the same number of controls (2D, organoids). Each condition was then imaged and/or pooled for downstream analysis (RNA, protein, flow cytometry, etc.). This was repeated at least three times for each patient. When comparing conditions across patients for drug testing, a minimum sample size of 5 repeats was used to account for interpatient variability. For all experiments, patients were chosen as representative of low, moderate and high chemoresistance based on in vivo data.
Data exclusions	No data have been excluded.
Replication	At least three independent replicates for each experiment were performed successfully.
Randomization	All samples compared in this study were patient-matched, therefore randomization was not necessary.
Blinding	During data acquisition and analysis data groups were labelled numerically to prevent investigator bias.

Behavioural & social sciences study design

All studies must disclose on these points even when the disclosure is negative.

Study description	*Briefly describe the study type including whether data are quantitative, qualitative, or mixed-methods (e.g. qualitative cross-sectional, quantitative experimental, mixed-methods case study).*
Research sample	*State the research sample (e.g. Harvard university undergraduates, villagers in rural India) and provide relevant demographic information (e.g. age, sex) and indicate whether the sample is representative. Provide a rationale for the study sample chosen. For studies involving existing datasets, please describe the dataset and source.*
Sampling strategy	*Describe the sampling procedure (e.g. random, snowball, stratified, convenience). Describe the statistical methods that were used to predetermine sample size OR if no sample-size calculation was performed, describe how sample sizes were chosen and provide a rationale for why these sample sizes are sufficient. For qualitative data, please indicate whether data saturation was considered, and what criteria were used to decide that no further sampling was needed.*
Data collection	*Provide details about the data collection procedure, including the instruments or devices used to record the data (e.g. pen and paper, computer, eye tracker, video or audio equipment) whether anyone was present besides the participant(s) and the researcher, and whether the researcher was blind to experimental condition and/or the study hypothesis during data collection.*
Timing	*Indicate the start and stop dates of data collection. If there is a gap between collection periods, state the dates for each sample cohort.*
Data exclusions	*If no data were excluded from the analyses, state so OR if data were excluded, provide the exact number of exclusions and the rationale behind them, indicating whether exclusion criteria were pre-established.*
Non-participation	*State how many participants dropped out/declined participation and the reason(s) given OR provide response rate OR state that no participants dropped out/declined participation.*

Randomization	*If participants were not allocated into experimental groups, state so OR describe how participants were allocated to groups, and if allocation was not random, describe how covariates were controlled.*

Ecological, evolutionary & environmental sciences study design

All studies must disclose on these points even when the disclosure is negative.

Study description	*Briefly describe the study. For quantitative data include treatment factors and interactions, design structure (e.g. factorial, nested, hierarchical), nature and number of experimental units and replicates.*
Research sample	*Describe the research sample (e.g. a group of tagged Passer domesticus, all Stenocereus thurberi within Organ Pipe Cactus National Monument), and provide a rationale for the sample choice. When relevant, describe the organism taxa, source, sex, age range and any manipulations. State what population the sample is meant to represent when applicable. For studies involving existing datasets, describe the data and its source.*
Sampling strategy	*Note the sampling procedure. Describe the statistical methods that were used to predetermine sample size OR if no sample-size calculation was performed, describe how sample sizes were chosen and provide a rationale for why these sample sizes are sufficient.*
Data collection	*Describe the data collection procedure, including who recorded the data and how.*
Timing and spatial scale	*Indicate the start and stop dates of data collection, noting the frequency and periodicity of sampling and providing a rationale for these choices. If there is a gap between collection periods, state the dates for each sample cohort. Specify the spatial scale from which the data are taken*
Data exclusions	*If no data were excluded from the analyses, state so OR if data were excluded, describe the exclusions and the rationale behind them, indicating whether exclusion criteria were pre-established.*
Reproducibility	*Describe the measures taken to verify the reproducibility of experimental findings. For each experiment, note whether any attempts to repeat the experiment failed OR state that all attempts to repeat the experiment were successful.*
Randomization	*Describe how samples/organisms/participants were allocated into groups. If allocation was not random, describe how covariates were controlled. If this is not relevant to your study, explain why.*
Blinding	*Describe the extent of blinding used during data acquisition and analysis. If blinding was not possible, describe why OR explain why blinding was not relevant to your study.*

Did the study involve field work? ☐ Yes ☐ No

Field work, collection and transport

Field conditions	*Describe the study conditions for field work, providing relevant parameters (e.g. temperature, rainfall).*
Location	*State the location of the sampling or experiment, providing relevant parameters (e.g. latitude and longitude, elevation, water depth).*
Access & import/export	*Describe the efforts you have made to access habitats and to collect and import/export your samples in a responsible manner and in compliance with local, national and international laws, noting any permits that were obtained (give the name of the issuing authority, the date of issue, and any identifying information).*
Disturbance	*Describe any disturbance caused by the study and how it was minimized.*

Reporting for specific materials, systems and methods

We require information from authors about some types of materials, experimental systems and methods used in many studies. Here, indicate whether each material, system or method listed is relevant to your study. If you are not sure if a list item applies to your research, read the appropriate section before selecting a response.

Materials & experimental systems

n/a	Involved in the study	
☐	☒	Antibodies
☐	☒	Eukaryotic cell lines
☒	☐	Palaeontology and archaeology
☐	☒	Animals and other organisms
☐	☒	Human research participants
☒	☐	Clinical data
☒	☐	Dual use research of concern

Methods

n/a	Involved in the study	
☒	☐	ChIP-seq
☐	☒	Flow cytometry
☒	☐	MRI-based neuroimaging

Antibodies

Antibodies used

EpCAM (CD326) VU1D9, Alexa 488, Cell Signaling 5198S, IF (1:1000)
Ki-67 B126.1, Abcam ab8191 , IF (1:1000)
EpCAM (CD326) , Abcam ab71916 , IF (1:1000)
α-SMA 1A4 , FITC, Sigma-Aldrich F3777, IF (1:1000)
Vimentin EPR3776 , Abcam ab92547, IF, WB (1:1000)
Vinculin V284 , Sigma-Aldrich SAB4200080 , WB (1:2000)
EpCAM (CD326) 9C4 PE, BioLegend 324206 , FC (1:20)
PDGFRα (CD140a) αR1, Alexa 647 , BD Biosciences 562798 , FC (1:20)
Prominin (CD133) AC133, PE , Miltenyi Biotec 130-080-801 , FC (1:40)
CXCR4 (CD184) 12G5, APC , BioLegend 306510, FC (1:20)
CD68 PG-M1 , Dako M0876, IHC (1:50)
Hyaluronan HABP2 , Biotin, Merck 385911, IHC (1:100)
Fibronectin , Sigma-Aldrich F3648, IHC (1:600)
Periostin , Sigma-Aldrich HPA012306, IHC (1:200)
TGF-βI , Sigma-Aldrich HPA017019, IHC (1:700)
Versican , Sigma-Aldrich HPA004726 , IHC (1:250)
Ki-67 MIB-1 , Dako M7240, IHC (1:1000)
Cleaved caspase 3 5AIE , Cell Signaling 9664S, IHC (1:50)

Validation

All antibodies used in our study are commercially available and validated (see below). They were tested in house against positive and negative controls to determine the degree of specific and unspecific binding.

EpCAM (CD326) VU1D9, Alexa 488, Cell Signaling 5198S, IF (1:1000)
"This Cell Signaling Technology antibody is conjugated to Alexa Fluor® 488 fluorescent dye and tested in-house for direct flow cytometry and immunofluorescent analysis in human cells. Specificity / Sensitivity: EpCAM (VU1D9) Mouse mAb (Alexa Fluor® 488 Conjugate) detects endogenous levels of total EpCAM protein. Species Reactivity: Human"

Ki-67 B126.1, Abcam ab8191 , IF (1:1000)
"Tested applications: Suitable for: Flow Cyt, IHC-Fr, IHC-P, ICC/IF. Species reactivity: Reacts with: Human, Marmoset (common)"

EpCAM (CD326) , Abcam ab71916 , IF (1:1000)
"Tested applications: Suitable for: ICC/IF, IHC-P, WB. Species reactivity: Reacts with: Mouse, Rat, Human"

α-SMA 1A4 , FITC, Sigma-Aldrich F3777, IF (1:1000)
"Specificity: Monoclonal Anti-Actin, α-Smooth Muscle specifically recognizes the α-smooth muscle isoform of actin (42 kDa) by ELISA and immunoblotting. It does not react with the other major actin isoforms present in fibroblasts or epithelial cells (β and γ-cytoplasmic), striated muscle (α-sarcomeric), myocardium (α-myocardial), or γ-smooth muscle isoform. Application Monoclonal: Anti-Actin, α-Smooth Muscle - FITC antibody is suitable for: (...) immunocytochemistry (...) Immunocytochemistry was performed on smooth muscle cells from bovine aortas using the monoclonal anti-ACTA2 antibody. Cells were first grown on glass cover slips and fixed in 50% acetone/EtOH for 10 minutes at 4 degrees."

Vimentin EPR3776 , Abcam ab92547, IF, WB (1:1000)
"Tested applications: Suitable for: Flow Cyt (Intra), ICC/IF, WB, IHC-P. Species reactivity: Reacts with: Mouse, Rat, Human, African green monkey."

Vinculin V284 , Sigma-Aldrich SAB4200080 , WB (1:2000)
"Immunogen: purified human platelet vinculin. Application: Monoclonal Anti-Vinculin antibody produced in mouse has been used in western blotting. Anti-Vinculin antibody, Mouse monoclonal has been used: to probe blots in immunoblotting."

EpCAM (CD326) 9C4 PE, BioLegend 324206 , FC (1:20)
"Application: FC - Quality tested. Each lot of this antibody is quality control tested by immunofluorescent staining with flow cytometric analysis. For flow cytometric staining, the suggested use of this reagent is 5 µl per million cells in 100 µl staining volume or 5 µl per 100 µl of whole blood. Product data: Human colon carcinoma cell line HT29 was stained with CD326 (clone9C4) PE (filled histogram) or mouseIgG2b, κ PE isotype control (openhistogram)."

PDGFRα (CD140a) αR1, Alexa 647 , BD Biosciences 562798 , FC (1:20)
"Reactivity: QC Testing: Human. Flow cytometric analysis of CD140a expression on the human MG-63 cell line. Human MG-63 cells were stained with either Alexa Fluor® 647 Mouse Anti-Human CD140a (Cat. No. 562798; solid line histogram) or Alexa Fluor® 647 Mouse mIgG2a, κ Isotype Control (Cat. No. 557715; dashed line histogram)."

Prominin (CD133) AC133, PE , Miltenyi Biotec 130-080-801 , FC (1:40)
"Specificity: Cells were incubated with an excess of purified unconjugated CD133/1 (AC133) antibody followed by staining with fluorochrome-conjugated antibodies of other known clones against the same marker. Based on the fluorescence signal obtained, the clones were identified as recognizing completely overlapping (++), partially overlapping (+), or completely different epitopes (-) of the marker. Sensitivity: Flow cytometric comparison of different clones for CD133/1. Human peripheral blood mononuclear cells (PBMCs) were stained with CD133/1 antibodies and with a suitable counterstaining. As a control, CD133/1 antibody staining was omitted and

cells were measured in the same channels."

CXCR4 (CD184) 12G5, APC , BioLegend 306510, FC (1:20)
"Application: FC - Quality tested. Each lot of this antibody is quality control tested by immunofluorescent staining with flow cytometric analysis. For flow cytometric staining, the suggested use of this reagent is 5 µl per million cells in 100 µl staining volume or 5 µl per 100 µl of whole blood. Product data: Human peripheral bloodlymphocytes stained with 12G5 APC."

CD68 PG-M1 , Dako M0876, IHC (1:50)
"Monoclonal Mouse Anti-Human CD68, Clone PG-M1, is intended for use in immunohistochemistry (IHC). The antibody labels macrophages. Specificity: The antibody was clustered as anti-CD68 at the Fifth International Workshop and Conference on Human Leucocyte Differentiation Antigens held in Boston in 1993. The antibody labels COS-1 and WOP cells transfected with CD68 cDNA. Unlike other CD68 antibodies, which label both macrophages and myeloid cells, the PG-M1 antibody detects a fixative-resistant epitope on the macrophage-restricted form of the CD68 antigen."

Hyaluronan HABP2 , Biotin, Merck 385911, IHC (1:100)
"Affinity purified. Binds specifically and strongly to hyaluronic acid (≥ 2000 M.W.). Composed of two binding polypeptides, which are derived from N-terminal regions of the hyaluronic acid binding proteoglycan and linkage proteins. Useful for ELISA and histochemistry."

Fibronectin , Sigma-Aldrich F3648, IHC (1:600)
"Determined to be immunospecific for human fibronectin by immunofluorescent labeling of human fibroblast cell cultures, ELISA and immuno-blotting. In immunoblotting, a specific band of fibronectin at 220kDa is observed (another band at 94 kDa may be also be present) using human fibronectin.When used in immunoelectrophoresis, the antibody shows 1-2 arcs of precipitation versus normal human plasma.This product maybe used for immunohistochemical localization of fibronectin in normal, inflamed and neoplastic tissues, for detection of fibronectin on cultured cells and structure and function studies of fibronectins in human and animal body fluids, tissues and cells."

Periostin , Sigma-Aldrich HPA012306, IHC (1:200)
TGF-βI , Sigma-Aldrich HPA017019, IHC (1:700)
Versican , Sigma-Aldrich HPA004726 , IHC (1:250)
"Prestige Antibodies® are highly characterized and extensively validated antibodies with the added benefit of all available characterization data for each target being accessible via the Human Protein Atlas portal linked just below the product name at the top of this page. The uniqueness and low cross-reactivity of the Prestige Antibodies® to other proteins are due to a thorough selection of antigen regions, affinity purification, and stringent selection. Prestige antigen controls are available for every corresponding Prestige Antibody and can be found in the linkage section. Every Prestige Antibody is tested in the following ways: IHC tissue array of 44 normal human tissues and 20 of the most common cancer type tissues. Protein array of 364 human recombinant protein fragments."

Ki-67 MIB-1 , Dako M7240, IHC (1:1000)
"Immunogen: Human recombinant peptide corresponding to a 1002 bp Ki-67 cDNA fragment (6). See package insert for reference(s). Species: Mouse Anti-Human. Specificity: In Western blotting of lysates of the multiple myeloma cell line, IM-9, the MIB-1 antibody labels bands of 345 and 395 kDa, identical to the bands labeled by the original Ki-67 antibody. Furthermore, Western blotting and competitive binding experiments clearly demonstrate that MIB-1, like the original Ki-67 antibody, reacts with an epitope encoded by a 66 bp repetitive element in the Ki-67 gene. In IHC the MIB-1 and the Ki-67 antibodies provide identical staining patterns on serial tonsillar frozen sections. The MIB-1 antibody recognizes native Ki-67 antigen and recombinant fragments of the Ki-67 molecule."

Cleaved caspase 3 5AIE , Cell Signaling 9664S, IHC (1:50)
"Cleaved Caspase-3 (Asp175) (5A1) Rabbit mAb detects endogenous levels of the large fragment (17/19 kDa) of activated caspase-3 resulting from cleavage adjacent to Asp175. This antibody does not recognize full length caspase-3 or other cleaved caspases. Application: Immunofluorescence (Immunocytochemistry)"

Eukaryotic cell lines

Policy information about cell lines

Cell line source(s)	Primary cell lines were established in house from patient tissue.
Authentication	No authentication was required since these are de novo established lines.
Mycoplasma contamination	All lines were routinely tested for Mycoplasma contamination, yielding negative results.
Commonly misidentified lines (See ICLAC register)	No commonly misidentified cell lines were used in the study.

Palaeontology and Archaeology

Specimen provenance	*Provide provenance information for specimens and describe permits that were obtained for the work (including the name of the issuing authority, the date of issue, and any identifying information).*
Specimen deposition	*Indicate where the specimens have been deposited to permit free access by other researchers.*

Dating methods | *If new dates are provided, describe how they were obtained (e.g. collection, storage, sample pretreatment and measurement), where they were obtained (i.e. lab name), the calibration program and the protocol for quality assurance OR state that no new dates are provided.*

☐ Tick this box to confirm that the raw and calibrated dates are available in the paper or in Supplementary Information.

Ethics oversight | *Identify the organization(s) that approved or provided guidance on the study protocol, OR state that no ethical approval or guidance was required and explain why not.*

Note that full information on the approval of the study protocol must also be provided in the manuscript.

Animals and other organisms

Policy information about studies involving animals; ARRIVE guidelines recommended for reporting animal research

Laboratory animals	8-week old female immunodeficient NMRI:Foxn1nu/nu mice (Charles River, UK).
Wild animals	The study did not involve wild animals.
Field-collected samples	The study did not involve fieldwork.
Ethics oversight	All in vivo experiments were approved by the animal experimental ethics committee (Home Office Project license PPI 70/8129) and performed in accordance with the Guidelines for Ethical Conduct in the Care and Use of Animals. Patient-derived xenografts (PDXs) were produced under decree no. 107/2012-B and 108/2012-B by the Italian Ministry of Health based on the legislative decree 106/92 regarding the protection of animals used in scientific research.

Note that full information on the approval of the study protocol must also be provided in the manuscript.

Human research participants

Policy information about studies involving human research participants

Population characteristics	Age, gender and cancer diagnosis of the patients analysed is given in Supplementary Table 2.
Recruitment	Patients were recruited via the University and Hospital Trust of Verona, the Technical University of Munich and the Barts Cancer Institute (see below). The healthy volunteers were recruited internally within the university. Potential self-selection biases include higher education and other socioeconomic indicators, which may skew certain biological aspects from the overall population; these are likely to be minor and do not compromise any of the results or conclusions of the present study.
Ethics oversight	Patient samples were collected through the ARC-Net Biobank of the University and Hospital Trust of Verona approved by the Verona University Hospital Ethics Committee (Program 1885 protocol 52438 23/11/2010, program 2172 protocol 26773 23/05/2012). PSCs were isolated from PDAC patient-derived tissues collected at the Technical University of Munich and approved by the Faculty of Medicine Ethics Committee (ethical approval 5510/12), and monocytes were derived from the blood from healthy donors at the Barts Cancer Institute (REC reference 17/EE/0182). Informed consent was obtained from all participants.

Note that full information on the approval of the study protocol must also be provided in the manuscript.

Clinical data

Policy information about clinical studies

All manuscripts should comply with the ICMJEguidelines for publication of clinical research and a completedCONSORT checklist must be included with all submissions.

Clinical trial registration	*Provide the trial registration number from ClinicalTrials.gov or an equivalent agency.*
Study protocol	*Note where the full trial protocol can be accessed OR if not available, explain why.*
Data collection	*Describe the settings and locales of data collection, noting the time periods of recruitment and data collection.*
Outcomes	*Describe how you pre-defined primary and secondary outcome measures and how you assessed these measures.*

Dual use research of concern

Policy information about dual use research of concern

Hazards

Could the accidental, deliberate or reckless misuse of agents or technologies generated in the work, or the application of information presented in the manuscript, pose a threat to:

No	Yes	
☐	☐	Public health
☐	☐	National security
☐	☐	Crops and/or livestock
☐	☐	Ecosystems
☐	☐	Any other significant area

Experiments of concern

Does the work involve any of these experiments of concern:

No	Yes	
☐	☐	Demonstrate how to render a vaccine ineffective
☐	☐	Confer resistance to therapeutically useful antibiotics or antiviral agents
☐	☐	Enhance the virulence of a pathogen or render a nonpathogen virulent
☐	☐	Increase transmissibility of a pathogen
☐	☐	Alter the host range of a pathogen
☐	☐	Enable evasion of diagnostic/detection modalities
☐	☐	Enable the weaponization of a biological agent or toxin
☐	☐	Any other potentially harmful combination of experiments and agents

ChIP-seq

Data deposition

☐ Confirm that both raw and final processed data have been deposited in a public database such as GEO.

☐ Confirm that you have deposited or provided access to graph files (e.g. BED files) for the called peaks.

Data access links<br>*May remain private before publication.*	*For "Initial submission" or "Revised version" documents, provide reviewer access links. For your "Final submission" document, provide a link to the deposited data.*
Files in database submission	*Provide a list of all files available in the database submission.*
Genome browser session<br>(e.g. UCSC)	*Provide a link to an anonymized genome browser session for "Initial submission" and "Revised version" documents only, to enable peer review. Write "no longer applicable" for "Final submission" documents.*

Methodology

Replicates	*Describe the experimental replicates, specifying number, type and replicate agreement.*
Sequencing depth	*Describe the sequencing depth for each experiment, providing the total number of reads, uniquely mapped reads, length of reads and whether they were paired- or single-end.*
Antibodies	*Describe the antibodies used for the ChIP-seq experiments; as applicable, provide supplier name, catalog number, clone name, and lot number.*
Peak calling parameters	*Specify the command line program and parameters used for read mapping and peak calling, including the ChIP, control and index files used.*
Data quality	*Describe the methods used to ensure data quality in full detail, including how many peaks are at FDR 5% and above 5-fold enrichment.*
Software	*Describe the software used to collect and analyze the ChIP-seq data. For custom code that has been deposited into a community repository, provide accession details.*

Flow Cytometry

Plots

Confirm that:

- [x] The axis labels state the marker and fluorochrome used (e.g. CD4-FITC).
- [x] The axis scales are clearly visible. Include numbers along axes only for bottom left plot of group (a 'group' is an analysis of identical markers).
- [x] All plots are contour plots with outliers or pseudocolor plots.
- [x] A numerical value for number of cells or percentage (with statistics) is provided.

Methodology

Sample preparation	Cells were extracted by dissociating PA hydrogels, organoids, spheres and 2D monolayers in TrypLE Express (Gibco, USA) with a micropipette. Samples were normalised to 106 cells/mL, blocked with Flebogamma (Grifols, Spain) and incubated with primary antibodies. For cell cycle analysis, cells were fixed with 4% paraformaldehyde, permeabilised with 0.25% Triton X-100 and stained with DAPI (10 µg/mL).
Instrument	BD LSRFortessa
Software	Data acquisition was performed on BD FACSDiva; fcs files were then analysed on FlowJo v10.
Cell population abundance	Samples were analysed at the 10,000 positive event threshold, that is gating for 10,000 intact cells (FSC-A vs SSC-A).
Gating strategy	Debris was excluded by gating intact cells (FSC-A 50k to 250k; SSC-A 10k to 150k). Singles cells were then gated to exclude doublets (SSC-A vs SSC-H); in the cell cycle experiments, this was further verified by gating in the DAPI width vs area plot. Cancer stem cells were selected as CD133/CXCR4 double positive cells, having gated for isotypes and FMOs.

- [x] Tick this box to confirm that a figure exemplifying the gating strategy is provided in the Supplementary Information.

Magnetic resonance imaging

Experimental design

Design type	*Indicate task or resting state; event-related or block design.*
Design specifications	*Specify the number of blocks, trials or experimental units per session and/or subject, and specify the length of each trial or block (if trials are blocked) and interval between trials.*
Behavioral performance measures	*State number and/or type of variables recorded (e.g. correct button press, response time) and what statistics were used to establish that the subjects were performing the task as expected (e.g. mean, range, and/or standard deviation across subjects).*

Acquisition

Imaging type(s)	*Specify: functional, structural, diffusion, perfusion.*
Field strength	*Specify in Tesla*
Sequence & imaging parameters	*Specify the pulse sequence type (gradient echo, spin echo, etc.), imaging type (EPI, spiral, etc.), field of view, matrix size, slice thickness, orientation and TE/TR/flip angle.*
Area of acquisition	*State whether a whole brain scan was used OR define the area of acquisition, describing how the region was determined.*

Diffusion MRI ☐ Used ☐ Not used

Preprocessing

Preprocessing software	*Provide detail on software version and revision number and on specific parameters (model/functions, brain extraction, segmentation, smoothing kernel size, etc.).*
Normalization	*If data were normalized/standardized, describe the approach(es): specify linear or non-linear and define image types used for transformation OR indicate that data were not normalized and explain rationale for lack of normalization.*
Normalization template	*Describe the template used for normalization/transformation, specifying subject space or group standardized space (e.g. original Talairach, MNI305, ICBM152) OR indicate that the data were not normalized.*

Noise and artifact removal	*Describe your procedure(s) for artifact and structured noise removal, specifying motion parameters, tissue signals and physiological signals (heart rate, respiration).*
Volume censoring	*Define your software and/or method and criteria for volume censoring, and state the extent of such censoring.*

Statistical modeling & inference

Model type and settings	*Specify type (mass univariate, multivariate, RSA, predictive, etc.) and describe essential details of the model at the first and second levels (e.g. fixed, random or mixed effects; drift or auto-correlation).*
Effect(s) tested	*Define precise effect in terms of the task or stimulus conditions instead of psychological concepts and indicate whether ANOVA or factorial designs were used.*

Specify type of analysis: ☐ Whole brain ☐ ROI-based ☐ Both

Statistic type for inference (See Eklund et al. 2016)	*Specify voxel-wise or cluster-wise and report all relevant parameters for cluster-wise methods.*
Correction	*Describe the type of correction and how it is obtained for multiple comparisons (e.g. FWE, FDR, permutation or Monte Carlo).*

Models & analysis

n/a	Involved in the study
☐	☐ Functional and/or effective connectivity
☐	☐ Graph analysis
☐	☐ Multivariate modeling or predictive analysis

Functional and/or effective connectivity	*Report the measures of dependence used and the model details (e.g. Pearson correlation, partial correlation, mutual information).*
Graph analysis	*Report the dependent variable and connectivity measure, specifying weighted graph or binarized graph, subject- or group-level, and the global and/or node summaries used (e.g. clustering coefficient, efficiency, etc.).*
Multivariate modeling and predictive analysis	*Specify independent variables, features extraction and dimension reduction, model, training and evaluation metrics.*
